# The link between gut microbiome and Alzheimer's disease: From the perspective of new revised criteria for diagnosis and staging of Alzheimer's disease

**DOI:** 10.1002/alz.14057

**Published:** 2024-06-28

**Authors:** Yuan Liang, Congcong Liu, Manman Cheng, Lijie Geng, Jing Li, Wenying Du, Minfang Song, Nian Chen, Traore Aicha Noura Yeleen, Li Song, Xiaoni Wang, Ying Han, Can Sheng

**Affiliations:** ^1^ Department of Neurology The Affiliated Hospital of Jining Medical University Jining China; ^2^ Department of Respiratory Medicine The Affiliated Hospital of Jining Medical University Jining China; ^3^ Department of Radiology The People's Hospital of Yanzhou Jining China; ^4^ Department of Emergency The Affiliated Hospital of Jining Medical University Jining China; ^5^ Department of Neurology China‐Japan Friendship Hospital Beijing China; ^6^ Department of Neurology Sir Run Shaw Hospital School of Medicine Zhejiang University Hangzhou China; ^7^ Department of Neurology Xuanwu Hospital of Capital Medical University Beijing China; ^8^ Key Laboratory of Biomedical Engineering of Hainan Province School of Biomedical Engineering Hainan University Haikou China; ^9^ Center of Alzheimer's Disease Beijing Institute for Brain Disorders Beijing China; ^10^ National Clinical Research Center for Geriatric Disorders Beijing China

**Keywords:** Alzheimer's disease, amyloid, biomarker, criteria, gut microbiome, inflammation, neurodegeneration, tau

## Abstract

Over the past decades, accumulating evidence suggests that the gut microbiome exerts a key role in Alzheimer's disease (AD). The Alzheimer's Association Workgroup is updating the diagnostic criteria for AD, which changed the profiles and categorization of biomarkers from “AT(N)” to “ATNIVS.” Previously, most of studies focus on the correlation between the gut microbiome and amyloid beta deposition (“A”), the initial AD pathological feature triggering the “downstream” tauopathy and neurodegeneration. However, limited research investigated the interactions between the gut microbiome and other AD pathogenesis (“TNIVS”). In this review, we summarize current findings of the gut microbial characteristics in the whole spectrum of AD. Then, we describe the association of the gut microbiome with updated biomarker categories of AD pathogenesis. In addition, we outline the gut microbiome‐related therapeutic strategies for AD. Finally, we discuss current key issues of the gut microbiome research in the AD field and future research directions.

**Highlights:**

- The new revised criteria for Alzheimer's disease (AD) proposed by the Alzheimer's Association Workgroup have updated the profiles and categorization of biomarkers from “AT(N)” to “ATNIVS.”
- The associations of the gut microbiome with updated biomarker categories of AD pathogenesis are described.
- Current findings of the gut microbial characteristics in the whole spectrum of AD are summarized.
- Therapeutic strategies for AD based on the gut microbiome are proposed.

## 1 INTRODUCTION

Alzheimer's disease (AD), mainly characterized by episodic memory loss, executive dysfunction, language impairment, and declined daily living ability, is the most common form of neurodegenerative disorder causing dementia.^1^ Currently, ≈ 6.7 million Americans aged ≥ 65 suffer from AD. By 2060, the number of individuals with AD dementia is projected to reach 13.8 million.^2^ The core pathological features of AD are extracellular amyloid beta (Aβ) deposition and intracellular neurofibrillary tangles derived from tau protein hyperphosphorylation.^3^ Currently, most of the interventions for AD focus on targeting Aβ aggregation and clearance.^4^, ^5^ Although some findings of phase II/III clinical trials are promising in terms of anti‐Aβ therapy,^6^, ^7^, ^8^, ^9^, ^10^ these investigations are still in the early stage, and need further validation for their long‐term therapeutic effect and safety. In addition, numerous pathological mechanisms and risk factors (e.g., inflammation, mitochondrial dysfunction, obesity, loss of hearing) may be closely associated with the onset of AD, suggesting the potential of multi‐target and comprehensive therapeutic strategies for AD.^1^, ^11^ Therefore, exploring novel mechanisms of AD is increasingly attracting researchers’ interest worldwide. In recent years, a growing number of studies have shown a close correlation between gut microbiome dysfunction and AD.^12^, ^13^, ^14^

Intestinal microbiome refers to the microbial community colonizing the human and animal gut, including bacteria, fungi, viruses, and so on. Among them, bacteria account for the highest proportion, with four top phyla, including *Proteobacteria*, *Firmicutes*, *Actinobacteria*, and *Bacteroidetes*.^15^ Alterations of gut microbial compositions and diversity are regarded as “gut microbial dysbiosis.” Current studies have found that dysbiosis in gut microbiota is linked to AD pathophysiological changes, such as abnormal brain Aβ aggregation, inflammation response, immune dysfunction, and neuronal and synaptic damage.^16^, ^17^, ^18^, ^19^ The impact of dysbiosis in gut microbiota on AD pathogenesis may be through the “microbiota–gut–brain” axis, which is a bidirectional communication pathway possibly mediated by the autonomic nervous system (ANS), neuroimmunity, enteroendocrine, short‐chain fatty acids (SCFA), neurotransmitters, and so on.^20^ Remarkedly, gut microbiome abnormalities have been observed in the whole clinical spectrum of AD, and have shown the potential for early identification of AD,^21^, ^22^, ^23^, ^24^, ^25^, ^26^ suggesting that dysbiosis in gut microbiota may serve as a valuable biomarker of AD. However, the regulatory mechanism of gut microbiota on AD pathogenesis remains largely unclear.

According to the Alzheimer's Association (AA) revised criteria for diagnosis and staging of AD, categorization of biomarkers has been updated, which includes: (1) core AD biomarkers (“A”: Aβ proteinopathy; “T”: tau proteinopathy); (2) non‐specific biomarkers involved in AD pathophysiology (“N”: injury, dysfunction, or degeneration of neuropil; “I”: inflammation, astrocytic activation), and (3) biomarkers of common non‐AD co‐pathologies (“V”: vascular brain injury; “S”: α‐synuclein). Notably, most of the previous studies focused on investigating the correlation between gut microbial alterations and amyloidosis (one of the core AD biomarkers), while few studies summarized the association of gut microbiota with other AD‐related pathological biomarkers. Accumulating evidence suggests that gut microbiome may also affect other AD‐related pathophysiological changes, such as tauopathy, inflammation/immune response, brain atrophy, regional glucose metabolism, and so on. Thus, understanding the impact of gut microbiota on different AD biomarker categories may help to comprehensively elucidate the “gut–brain” communication pathways in AD, which may further provide multiple potentially targeted interventions for AD.

In this review, we first summarize the evolution of diagnostic criteria for AD. Then, we review current findings of gut microbiome characteristics in different clinical stages of AD, including preclinical AD, mild cognitive impairment (MCI), and AD dementia (Table 1). Additionally, the correlations between the gut microbiota and the updated three broad biomarker categories of AD pathogenesis are discussed. Furthermore, we discuss the microbiome‐mediated therapeutic strategies for AD. Finally, we conclude with current issues of the gut microbiome research in AD and highlight future perspectives of this field. The aim of this review is: (1) to clarify the pathophysiological mechanisms of AD from the perspective of the “microbiota–gut–brain” axis, and (2) to explore novel therapeutic strategies for AD.

**TABLE 1:** Summary of altered gut microbiome in human AD.

Study	Year	Participants	Criteria for AD	Analysis approach	Main findings
Vogt et al.^25^	2017	AD (*n* = 25) Control (*n* = 25)	Clinical definition	16S rRNA amplicon sequencing	AD patients showed decreased *Firmicutes* and *Bifidobacterium* and increased *Bacteroidetes* compared to healthy controls.
Cattaneo et al.^19^	2017	Cognitively impaired patients with brain amyloidosis (*n* = 40); cognitively impaired patients with no brain amyloidosis (*n* = 33); control (*n* = 10)	Biomarker definition	Microbial DNA qPCR Assay Kit	Amyloid positive individuals had lower anti‐inflammatory *Eubacterium rectale* and higher inflammatory *Escherichia/Shigella* than both healthy controls and amyloid negative individuals.
Manderino et al.^27^	2017	Cognitively intact group (*n* = 25); cognitively impaired group (*n* = 18)	Clinical definition	16S rRNA amplicon sequencing	Cognitively intact group exhibited a lower proportion of *Bacteroidetes* and *Proteobacteria* and higher proportions of *Firmicutes* and *Verrucomicrobia* than the cognitively impaired group.
Zhuang et al.^28^	2018	AD (*n* = 43); control (*n* = 43)	Clinical definition (*in subgroup analysis, 12 subjects underwent amyloid PET)	16S rRNA amplicon sequencing	AD patients showed a decrease in *Bacteroidetes* and an increase in *Actinobacteria* in AD patients compared to healthy controls.
Liu et al.^23^	2019	AD (*n* = 33); aMCI (*n* = 32); control (*n* = 32)	Clinical definition	16S rRNA amplicon sequencing	AD patients showed reduced *Firmicutes* and increased *Proteobacteria* compared to controls. *Gammaproteobacteria*, *Enterobacteriales*, and *Enterobacteriaceae* showed a progressive enriched prevalence from controls to aMCI and AD patients.
Saji et al.^29^	2019	Demented (*n* = 34); non‐Demented (*n* = 94)	Clinical definition	16S rRNA amplicon sequencing	Demented had reduced *Bacteroides* and the *Firmicutes/Bacteroidetes* ratio compared to non‐demented patients.
Li et al.^24^	2019	AD (*n* = 30); aMCI (*n* = 30); control (*n* = 30)	Clinical definition	16S rRNA amplicon sequencing	MCI patients showed similar gut microbial alterations like AD patients.
Sheng et al.^26^	2021	CI (*n* = 14); SCD (*n* = 53); control (*n* = 38)	Clinical definition (*in subgroup analysis, 59 subjects underwent amyloid PET)	16S rRNA amplicon sequencing	The abundance of the anti‐inflammatory genus *Faecalibacterium* was significantly decreased in SCD compared to controls.
Liu et al.^30^	2021	aMCI (*n* = 20); control (*n* = 22)	Clinical definition	16S rRNA amplicon sequencing	Phylum *Bacteroidetes* was higher in aMCI than that in controls, while no significant difference in *Firmicutes* and *Proteobacteria* was found.
Guo et al.^31^	2021	AD (*n* = 18); MCI (*n* = 20); control (*n* = 18)	Clinical definition	16S rRNA amplicon sequencing	Patients newly diagnosed with AD or MCI had decreased *Bacteroides*, *Lachnospira*, and *Ruminiclostridium* 9 and increased *Prevotella* at the genus level.
Pan et al.^32^	2021	MCI (*n* = 22); control (*n* = 26)	Clinical definition	16S rRNA amplicon sequencing	The relative abundance of *Bacteroidetes* was lower in MCI than in controls, while *Fusobacteria* were significantly more abundant in MCI than in controls.
Zhou et al.^33^	2021	AD (*n* = 60); control (*n* = 32)	Clinical definition	16S rRNA amplicon sequencing	*Bifidobacterium*, *Sphingomonas*, *Lactobacillus*, and *Blautia* were enriched, while *Odoribacter*, *Anaerobacterium*, and *Papillibacter* were reduced in AD. AD patients with NPS showed decreased *Chitinophagaceae*, *Taibaiella*, and *Anaerobacterium*.
Ling et al.^34^	2020	AD (*n* = 88); control (*n* = 65)	Clinical definition	ITS2 rRNA sequencing	*Candida tropicalis* and *Schizophyllum* commune were enriched in the AD patients, while *Rhodotorula mucilaginosa* decreased significantly.
Ling et al.^35^	2020	AD (*n* = 100); control (*n* = 71)	Clinical definition	16S rRNA amplicon sequencing	AD patients had a decrease in *Faecalibacterium* and an increase in *Bifidobacterium* compared to controls.
Sheng et al.^22^	2022	AD (*n* = 11); MCI (*n* = 11); CN+ (*n* = 32); CN− (*n* = 34)	Biomarker definition	16S rRNA amplicon sequencing	CN+ patients showed a decrease in *Firmicutes* and *Deltaproteobacteria* and an increase in *Bacteroidetes* compared to CN−. The combination of plasma Aβ and altered gut microbiota had the potential in identifying CN+.
Cirstea et al.^36^	2022	AD (*n* = 45); control (n = 54)	Clinical definition	16S rRNA amplicon sequencing	The gut microbiota of AD patients in a Canadian cohort was not overtly different from controls, while the oral microbiota displayed marked differences.
He et al.^37^	2023	CI (*n* = 30); SCD (*n* = 62); control (*n* = 35)	Clinical definition	16S rRNA amplicon sequencing	The abundance of *Lachnospiraceae*, *Fusicatenibacter*, *Lachnospiracea_incertae_sedis*, and *Anaerobutyricum* decreased with cognitive ability. *Rikenellaceae*, *Alistipes*, and *Odoribacteraceae* were specifically enriched in the CI group.
Ferreiro et al.^21^	2023	Preclinical AD (*n* = 49); control (*n* = 115)	Biomarker definition	Metagenomic sequencing	*Dorea formicigenerans*, *Oscillibacter sp. 57_20*, *Faecalibacterium prausnitzii*, *Coprococcuscatus*, and *Anaerostipes hadrus* were most associated with preclinical AD status. Altered gut microbiome profiles correlated with Aβ and tau, but not neurodegeneration.

Abbreviations: Aβ, amyloid beta; AD, Alzheimer's disease; aMCI, amnestic mild cognitive impairment; CI, cognitive impairment; CN‐, amyloid beta–negative cognitively normal; CN+, amyloid beta–positive cognitively normal; MCI, mild cognitive impairment; NPS, neuropsychiatric symptoms; PET, positron emission tomography; qPCR, quantitative polymerase chain reaction; SCD, subjective cognitive decline.

## 2 THE EVOLUTION OF DIAGNOSTIC CRITERIA FOR AD

The diagnostic criteria for AD have been evolving during the past decades. The first clinical diagnosis of AD was established by the National Institute of Neurological and Communicative Disorders and Stroke (NINCDS) and the Alzheimer's Disease and Related Disorders Association (ADRDA) in 1984.^38^ The definition of AD is mainly based on clinical symptoms and histopathologic evidence obtained from brain biopsy or autopsy. With the advances of available AD biomarkers in vivo, such as structural magnetic resonance imaging (MRI), molecular neuroimaging with positron emission tomography (PET), and cerebrospinal fluid (CSF) analysis of Aβ or tau proteins, the International Working Group (IWG) proposed a new conceptual framework for the diagnosis of AD in 2007, which highlighted the combination of clinical phenomenology and biological markers.^39^ In 2014, the IWG further divided biomarkers into two categories: diagnostic markers (Aβ_1‐42_, total tau [t‐tau], phosphorylated tau [p‐tau] in CSF, amyloid PET), and progression markers (structural MRI and fluorodeoxyglucose [FDG] PET].^40^

In 2011, the National Institute on Aging and the Alzheimer's Association (NIA‐AA) published separate recommendations for the diagnosis of AD in its preclinical, MCI, and dementia states.^41^, ^42^, ^43^ These criteria also proposed a model of the pathophysiological sequence of AD, of which Aβ accumulation is an “upstream” event in the cascade leading to the “downstream” synaptic dysfunction, neurodegeneration, and eventual neuronal loss. In 2018, the NIA‐AA further categorized biomarkers into Aβ deposition, pathologic tau, and neurodegeneration [AT(N)].^44^ If an individual has an abnormal Aβ deposition evidence, but a biomarker for tau is not available, then the individual is placed into the “Alzheimer's continuum.” The diagnostic framework is a purely biological definition of AD that relies on biomarkers.

Due to the occurrence of major developments in AD, the 2018 NIA‐AA research framework has been further updated (see https://aaic.alz.org/nia‐aa.asp). In addition to traditional CSF and imaging biomarkers, the present criteria have incorporated plasma biomarkers into updated biomarker categorization, disease diagnosis, and staging. This full multimodal biomarker profile is further categorized as ATNX. “X” is added to the new biomarker categorization and refers to inflammation (I), vascular brain injury (V), and α‐synuclein (S). The revised guidelines highlight that biological diagnosis and staging of AD is now transitioning from the purpose of research to the application in clinical practice. The progression of diagnostic criteria for AD is briefly summarized in Table 2.[Fig alz14057-fig-0001]

**TABLE 2:** The progression of diagnostic criteria for AD (1984–2023).

	1984^38^	2007^39^	2011^41^, ^43^	2014^40^	2018^44^	2023*
**Working group**	NINCDS–ADRDA	IWG	NIA‐AA	IWG‐2	NIA‐AA	AA
**Key diagnostic points**	**Probable AD**: Dementia syndrome *PLUS* clinical features of the AD phenotype **Definite AD**: Clinical criteria for probable AD *PLUS* histopathological evidence	**Probable AD**: Early episodic memory impairment *PLUS* one or more supportive features (MTA, abnormal CSF biomarker, specific pattern on PET) **Definite AD**: Clinical phenotype *PLUS* histopathological or genetic evidence	**Preclinical AD**: Stage 1: asymptomatic amyloidosis Stage 2: asymptomatic amyloidosis *PLUS* neurodegeneration Stage 3: amyloidosis *PLUS* neurodegeneration *PLUS* subtle cognitive decline **MCI due to AD**: Clinical criteria (objective impairment in cognitive domains, preservation of daily functional abilities and no dementia) *PLUS* Aβ (PET or CSF) *PLUS* neuronal injury (CSF tau, FDG PET, structural MRI) **Probable AD dementia**: Clinical criteria *PLUS* Aβ (PET or CSF) *PLUS* neuronal injury (CSF tau, FDG PET, structural MRI)	**Typical AD**: Early episodic memory impairment *PLUS* in vivo evidence of AD pathology (abnormal CSF biomarker, amyloid PET, genetic evidence in PSEN1, PSEN2, or APP) **Atypical AD**: Specific clinical phenotype (posterior variant, logopenic variant, frontal variant, and Down's syndrome variant of AD) *PLUS* in vivo evidence of AD pathology (abnormal CSF biomarker, amyloid PET, genetic evidence in PSEN1, PSEN2, or APP)	**Biomarker profiles and categories**: (1) Normal: –T–(N)– (2) AD continuum: A+T–(N)–, A+T+(N)–, A+T+(N)+, A+T–(N)+ (3) Non‐AD pathologic change: A–T+(N)–, A–T–(N)+, A–T+(N)+ **Numeric clinical staging in the AD continuum**: Stage 1: asymptomatic Stage 2: transitional decline: subjective cognitive decline or mild neurobehavioral changes Stage 3: MCI Stage 4: mild dementia Stage 5: moderate dementia Stage 6: severe dementia	**Biological staging with PET**: Stage a (*initial*): A+T–; Stage b (*early*): A+T_MTL_+; Stage c (*intermediate*): A+T_MOD_+; Stage d (*advanced*): A+T_HIGH_+ **Numeric clinical staging in the AD continuum**: Stage 0: asymptomatic, AD gene Stage 1: asymptomatic, biomarker evidence only Stage 2: transitional decline Stage 3: MCI Stage 4: mild dementia Stage 5: moderate dementia Stage 6: severe dementia
**Biomarkers**	No biomarker	**No classification of all biomarkers**: 1. Structural MRI 2. CSF Aβ_1–42_, t‐tau, p‐tau 3. FDG PET or amyloid PET	**Biomarkers of Aβ deposition**: CSF Aβ42, PET amyloid imaging **Biomarkers of neuronal injury**: CSF tau/p‐tau, structural MRI, FDG PET imaging, SPECT perfusion imaging, and other measures (fMRI, DTI, etc.) **Other associated biomarkers**: inflammatory, oxidative stress, synaptic damage biomarkers	**Diagnostic biomarkers**: CSF (low Aβ_1–42_ and high t‐tau or p‐tau), amyloid PET **Progression biomarkers**: Structural MRI, FDG PET	**AT(N) profiles**: A: CSF Aβ_42_, or Aβ_42_/Aβ_40_ ratio, amyloid PET T: CSF p‐tau, tau PET N: anatomic MRI, FDG PET, CSF t‐tau	**Core Biomarkers**: A: fluid Aβ_42_, amyloid PET T: fluid p‐tau 181, 217, p‐tau 231, MTBR‐243, non‐phosphorylated tau, tau PET **Non‐specific biomarkers**: N: fluid NfL, anatomic MR, FDG PET I: fluid GFAP **Non‐AD co‐pathology biomarkers**: V: anatomic infarction, WMH S: CSF αSyn‐SAA

Abbreviations: AA, Alzheimer's Association; Aβ, amyloid beta; AD, Alzheimer's disease; APP, amyloid precursor protein; αSyn‐SAA, alphaSyn seed amplification assay; CSF, cerebrospinal fluid; DTI, diffusion tensor imaging; FDG PET, fluorodeoxyglucose positron emission tomography; fMRI, functional magnetic resonance imaging; GFAP, glial fibrillary acidic protein; IWG, International Working Group; MCI, mild cognitive impairment; MRI, magnetic resonance imaging; MTA, medial temporal atrophy; MTBR, microtubule binding region; NfL, neurofilament light chain; NIA‐AA, National Institute on Aging–Alzheimer's Association; NINCDS–ADRDA, National Institute of Neurological and Communicative Disorders and Stroke–Alzheimer's Disease and Related Disorders Association; PET, positron emission tomography; p‐tau, phosphorylated tau; PSEN1, Presenilin 1; PSEN2, Presenilin 2; SPECT, single photon emission computed tomography; t‐tau, total‐tau; WMH, white matter hyperintensity.

* https://aaic.alz.org/nia‐aa.asp.

## 3 ALTERATIONS OF THE GUT MICROBIOTA IN AD

Currently, specific alterations in the composition and diversity of the gut microbiota have been found in different clinical stages of AD.^21^, ^23^, ^24^, ^25^, ^26^, ^27^, ^28^, ^30^, ^34^, ^35^ Vogt et al. reported significant differences in the gut microbial composition of AD patients compared to healthy controls, with significantly reduced *Firmicutes* and *Bifidobacterium*, and increased *Bacteroidetes* in AD.^25^ Liu et al. also confirmed the decreased fecal microbial diversity and proportion of *Firmicutes* in AD patients compared to healthy subjects.^23^ Additionally, they first found a progressive enriched prevalence of *Gammaproteobacteria*, *Enterobacteriales*, and *Enterobacteriaceae* from controls to amnestic MCI (aMCI) and AD patients.^23^

Subjective cognitive decline (SCD), characterized by a self‐reported decline in the memory and/or other cognitive domains without objective cognitive impairment, is considered the earliest clinical symptom of preclinical AD.^45^ Current research has shown that individuals with SCD exhibit specific gut microbial profiles similar to patients with AD.^26^ The abundance of phylum *Firmicutes* and its corresponding *Clostridia*, *Clostridiales*, *Ruminococcaceae*, and *Faecalibacterium* also showed a trend toward a progressive decline from controls to SCD individuals and cognitive impairment patients. Specifically, the abundance of the anti‐inflammatory genus *Faecalibacterium* was significantly decreased in SCD compared to controls, suggesting that alterations of gut microbial composition may be present in preclinical AD. Interestingly, a few studies have also shown that gut microbiome composition may serve as an indicator of preclinical AD. For instance, one study reported that the relative abundance of phylum *Bacteroidetes* was significantly enriched, whereas phylum *Firmicutes* and class *Deltaproteobacteria* were significantly decreased in Aβ‐positive cognitively normal individuals compared to that in Aβ‐negative cognitively normal individuals.^22^ Using metagenomic sequencing analysis, Ferreiro et al.^21^ further identified some species most associated with preclinical AD status, including *Dorea formicigenerans*, *Oscillibacter sp. 57_20*, *Faecalibacterium prausnitzii*, *Coprococcus catus*, and *Anaerostipes hadrus*. Currently, a systematic review summarizes the gut microbiome characteristics in SCD, MCI, and AD patients. They found that the relative abundance of phylum *Firmicutes* was significantly lower in AD and MCI than controls, while the relative abundance of phylum *Bacteroidetes* was significantly higher in MCI than controls. An increasing trend for *Enterobacteriaceae* and a decreasing trend for *Ruminococcaceae*, *Lachnospiraceae*, and *Lactobacillus* were present during AD.^46^

However, several studies reported contradictory results.^29^, ^32^, ^33^, ^36^ Zhuang et al. found that the abundance of *Bacteroidetes* in AD patients significantly decreased, while the abundance of *Ruminococcaceae*, *Enterococcaceae*, and *Lactobacillus* significantly increased.^28^ Another study also showed that patients with AD had decreased *Bacteroides*.^31^ These discrepancies in gut taxonomic compositions among different studies may be due to the heterogeneity in severity of illness, different diagnostic criteria, racial difference, lifestyle, comorbidities, medications, and so on. Thus, a standardized study protocol is necessary for AD gut microbiome in the future.

In summary, most studies have suggested that the gut microbiota in different clinical stages of AD has significant alterations. However, larger scale studies are still needed to validate current findings and determine the specific bacterial species associated with the precision diagnosis of AD.

## 4 THE CORRELATION BETWEEN THE GUT MICROBIOTA AND AD PATHOGENESIS

### 4.1 Gut microbiota and AD core biomarkers

#### 4.1.1 Gut microbiota and “A”

Accumulating evidence indicates that brain Aβ deposition is correlated with gut dysbiosis.^47^, ^48^, ^49^, ^50^, ^51^, ^52^, ^53^ A prior study found that genera identified as differentially abundant in AD were significantly associated with AD biomarkers in CSF, including the Aβ_42_/Aβ_40_ ratio, p‐tau, and the p‐tau/Aβ_42_ ratio.^25^ Sheng et al.^22^ also confirmed that based on Aβ PET scans, the global brain Aβ burden was negatively associated with family *Desulfovibrionaceae*, genus *Bilophila*, and genus *Faecalibacterium*. In addition, the causal relationship between the gut microbiota and AD pathophysiology has been investigated., et al.^54^ found that the transplantation of fecal microbiota from healthy wild‐type mice into transgenic AD mice ameliorated the formation of Aβ plaques and neurofibrillary tangles, glial reactivity and cognitive impairment, indicating that the pathogenesis of AD may be mediated by gut microbiota. Moreover, many studies have reported that antibiotic‐induced perturbations in gut microbial compositions and diversity can also influence amyloidosis in a mouse model of AD, leading to decreased cerebral Aβ deposition.^47^, ^48^, ^49^, ^50^, ^52^ Thus, these studies suggest a strong causal correlation between gut dysbiosis and AD pathology.

**FIGURE 1 alz14057-fig-0001:**
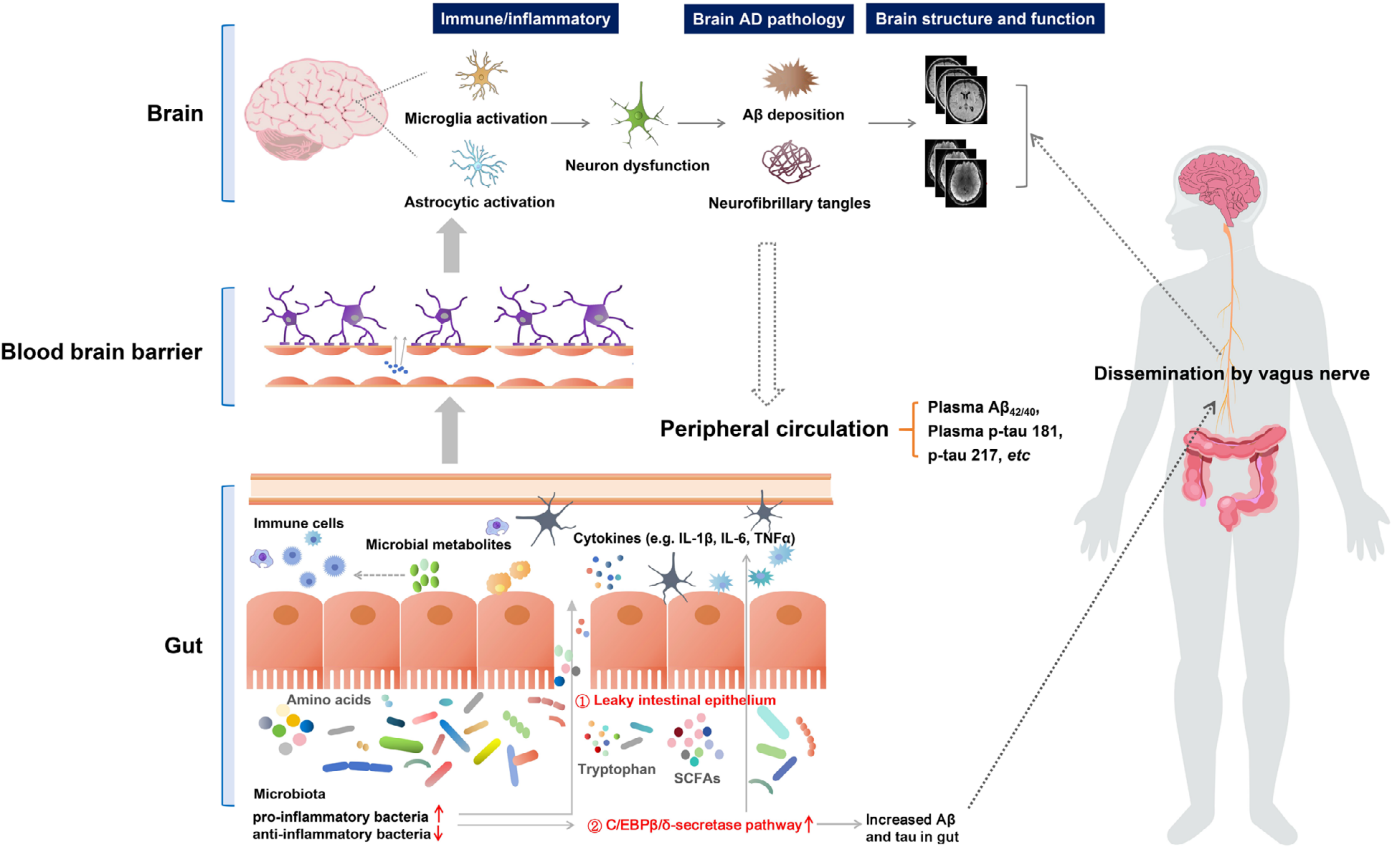
The association of the gut microbiota with AD‐related pathogenesis. Significant alterations in the gut microbial composition are observed in AD, with increased pro‐inflammatory bacteria and decreased anti‐inflammatory bacteria. Two pathways will lead to the activated inflammatory response in the peripheral circulation: (1) Abnormal gut microbiota exacerbates the permeability of intestinal epithelium, leading to the release of several cytokines (e.g., IL‐1β, IL‐6, TNFα), chemokines, and microbial metabolites. These substances may further infiltrate the blood and lymphatic system. (2) Gut dysbiosis contributes to the C/EBPβ/AEP (δ‐secretase) signaling activation, which also induces the release of cytokines. In addition, the activated C/EBPβ/AEP pathway prompts the Aβ and tau pathology in the gut, which will further be disseminated to the brain via the vagus nerve and exacerbate AD‐related pathology. These pro‐inflammatory substances in the peripheral circulation can permeate into the brain through the damaged BBB. In the brain, the activation of microglia and astrocytes contributes to neuron dysfunction, accelerating the AD pathology, which further leads to the disruption of brain structure and function. Aβ, amyloid beta; AD, Alzheimer's disease; AEP, asparagine endopeptidase; BBB, blood–brain barrier; IL, interleukin; p‐tau, phosphorylated tau; SCFA, short‐chain fatty acid; TNFα, tumor necrosis factor alpha.

The potential mechanisms of the gut microbiota‐mediated AD pathogenesis may be as follows: first, abnormal gut microbiota–host interactions may exacerbate the permeability of the intestinal epithelium, leading to the release of cytokines, chemokines, neurotransmitters, and gut‐derived metabolites that infiltrate the blood and lymphatic system.^20^ Additionally, gut dysbiosis can also lead to the damage of the blood–brain barrier (BBB). Thus, these circulating substances can permeate into the brain through the damaged BBB, accelerating AD pathology.^55^, ^56^ Second, gut dysbiosis contributes to amyloid pathology via the C/EBPβ/asparagine endopeptidase (AEP) signaling activation. C/EBPβ, as an inflammatory cytokine or Aβ‐activated transcription factor, regulates the expression of AEP. AEP, also as a δ‐secretase, can cleave both Aβ precursor protein (APP) and tau, promoting the formation of Aβ and neurofibrillary tangles. Current studies have shown that C/EBPβ/AEP signaling is activated by gut dysbiosis in mouse models of AD, whereas C/EBPβ/AEP signaling and arachidonic acid–associated inflammatory enzymes are diminished in germ‐free mice. Therefore, the C/EBPβ/AEP pathway plays a critical role in the impact of gut microbiota on AD pathologies. Finally, inflammatory response and glial reactivity may participate in the influence of gut microbial dysbiosis on AD pathogenesis.^50^, ^57^, ^58^ Peripheral inflammatory mediators can infiltrate the brain, and then activate the Toll‐like receptors (TLR), such as TLR2/1, and nuclear factor kappa‐B (NF‐κB) signaling pathways, leading to brain inflammatory responses.^59^ In this review, the association of the gut microbiota with AD pathogenesis is shown in Figure 1.

#### 4.1.2 Gut microbiota and “T”

Gut dysbiosis is reported to be associated with tau pathological biomarkers. One study investigated the correlation between altered gut microbiota and AD‐related biomarkers among patients with AD, MCI, and SCD. The results showed that declined SCFA‐producing microbiota, including *Lachnospiraceae spp*., *Lachnoclostridium spp*., *Roseburia hominis*, and *Bilophila wadsworthia*, was associated with higher odds of positive CSF p‐tau status.^60^ Using PET tau imaging, Ferreiro et al.^21^ also found that gut microbiome profiles correlated with tau biomarkers in cognitively normal individuals. However, the correlation between gut microbiota and specific tau proteins in CSF and plasma, such as p‐tau 181 and p‐tau 217, is still largely unclear.

The misfolding and aggregation of tau is a key hallmark of AD. Researchers have shown that DNA extracted from bacteria, especially certain bacterial species associated with AD (*B. burgdorferi*, *P. gingivalis*, *C. albicans*, and *E. col*) promotes pronounced tau aggregation,^61^ suggesting that microbial DNA may play an important role in the tau protein misfolding and AD pathogenesis. Notably, some strains of *E. coli* (such as K99) and *P. gingivalis* have also been identified in the brain of patients with AD.^62^, ^63^ These strains have properties of facultative intracellular parasites, contributing to the interaction of bacterial DNA with tau proteins inside the neuron. These bacterial DNA can be secreted into the outer membrane and released into the neuron's cytosol, thus becoming the seed for tau aggregation.^61^ The findings provide new insights into the relationship between gut microbiota and tau protein aggregation, opening novel opportunities for AD therapeutic interventions.

### 4.2 Gut microbiota and non‐specific biomarkers involved in AD pathophysiology

#### 4.2.1 Gut microbiota and “N”

In the new, revised criteria for AD, neurodegenerative biomarkers (“N”) refers to neuroimaging markers (anatomic MR, FDG PET) and fluid neurofilament light chain (NfL). Several studies have confirmed the regional brain atrophy, such as hippocampus, medial temporal lobe, and so on, in patients with AD and those who have a high risk for conversion to AD dementia. Currently, the effect of gut microbiota on brain anatomic changes has attracted wide interest from researchers.^64^ Zhu et al.^65^ investigated the association of gut microbiota with structural MRI measures in 157 healthy young adults. They found that gut microbial diversity was negatively correlated with gray matter volume (GMV) within the prefrontal, parietal, temporal, occipital, cingulate cortices, and insula. Notably, the gut–brain interactions may be sex dependent.^65^, ^66^ In addition, they also reported that gut microbial diversity and enterotypes could indirectly impact cognitive performance by mediating the topological properties of structural networks, such as small‐worldness.^67^ In a larger community‐based cohort of 1430 participants, the relative abundance of *Odoribacter* was found to be positively associated with the right hippocampal volume.^68^ However, another study showed that there was no significant correlation between gut microbiome profiles and hippocampus volume in a cohort of cognitively normal older adults.^21^ The discrepancy in these results may be due to the age range, race, and cognitive performance. He et al. first studied the association of gut microbiota with brain structural changes in the spectrum of AD, including controls, SCD, and cognitive impairment (CI) participants.^37^ They found that the relative abundance of *Mediterraneibacter* was significantly correlated with changes in brain GMV and regional cortical structures, such as the internal olfactory area and the parahippocampal gyrus.^37^ Moreover, in a 24‐week randomized, double‐blind, placebo‐controlled trial, older MCI patients with probiotic *Bifidobacterium breve* consumption exhibited suppressed brain atrophy progression,^69^ indicating a direct impact of modifying gut microbiota on brain structural changes.

**FIGURE 2:**
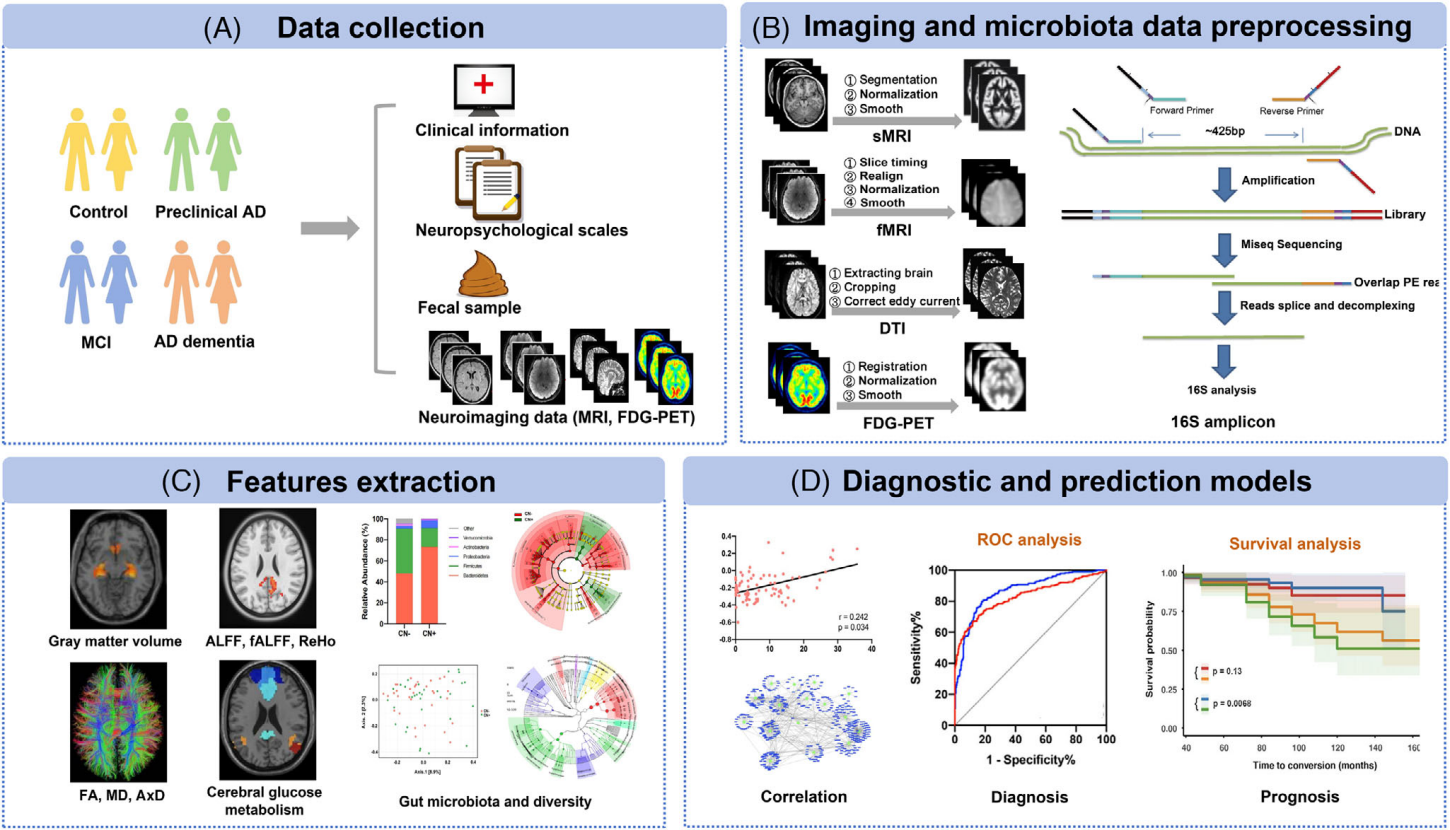
The framework of *radiomicrobiomics* research in AD. A, Data collection: recruitment of healthy controls and the spectrum of AD, including preclinical AD, MCI, and AD dementia. Clinical information, neuropsychological scales, fecal sample, and multi‐modal neuroimaging scan data (MRI and FDG PET) are collected. B, Imaging and microbiota data preprocessing: image preprocessing of sMRI, fMRI, DTI, and FDG PET; 16S high‐throughput sequencing. C, Features extraction: key neuroimage (e.g., gray matter volume, ALFF, fALFF, ReHo, FA, MD, AxD, regional glucose metabolism) and microbial features (e.g., microbial flora, α‐diversity, β‐diversity) are extracted. D, Diagnostic and prediction models: the correlation analysis is used to investigate the relationship between different features and clinical assessments. Based on the selected key multi‐omics features, the ROC analysis and survival analysis are separately used for the establishment of diagnostic model and prediction model. AD, Alzheimer's disease; AxD, axial diffusion; ALFF, amplitude of low‐frequency fluctuation; DTI, diffusion tensor imaging; FA, fractional anisotropy; fALFF, fractional amplitude of low‐frequency fluctuation; FDG PET, fluorodeoxyglucose positron emission tomography; fMRI, functional magnetic resonance imaging; MCI, mild cognitive impairment; MD, mean diffusivity; MRI, magnetic resonance imaging; ReHo, regional homogeneity; ROC, receiver operating characteristic curve; sMRI, structural magnetic resonance imaging.

Brain glucose hypometabolism is a hallmark of AD. FDG PET, as a well‐established technique to invasively quantify the resting‐state cerebral glucose metabolic level in vivo, has been used to elucidate the abnormal brain glucose uptake in AD and predict the progression of cognitive impairment. Gut microbiota is also considered to play a role in regulating glucose metabolism.^70^, ^71^, ^72^ Current evidence supports that the gut microbiome can impact glucose and energy homeostasis by regulating host gut–brain signaling and initiating direct communication to the brain via microbe‐derived metabolites.^71^ Thus, FDG PET may mirror the effect of gut microbiota on brain glucose metabolism. For instance, using 2‐deoxy‐2‐[^18^F] FDG‐PET, one study revealed that C57BL/6 J mice treated with 27‐hydroxycholesterol altered gut microbial compositions and decreased brain glucose uptake value, finally inducing memory impairment.^73^ This study suggests that reduced brain glucose uptake was mediated by the gut microbiota. However, studies involving the correlation between gut microbiota and FDG PET biomarkers are still lacking. Currently, the combination of the gut microbiota and neuroimaging techniques is called *radiomicrobiomics*. In this review, a summary of *radiomicrobiomics* studies is shown in Table 3 and the framework of *radiomicrobiomics* research is depicted in Figure 2.

**TABLE 3:** Summary of radiomicrobiomics studies in human AD.

Study	Year	Participants	Radiomicrobiomics techniques	Main findings
Saji et al.^64^	2019	MCI (*n* = 61); control (*n* = 21)	Structural MRI 16S rRNA amplicon sequencing	MCI patients with more *Bacteroides* were more likely to present with high cortical and hippocampal atrophy.
Saji et al.^29^	2019	Demented (*n* = 34); non‐demented (*n* = 94)	Structural MRI 16S rRNA amplicon sequencing	A lower prevalence of *Bacteroides* and a higher prevalence of “other” bacteria was associated with higher odds ratios than the traditional dementia biomarkers like high VSRAD score.
Liu et al.^30^	2021	MCI (*n* = 20); control (*n* = 22)	Resting‐state functional MRI 16S rRNA amplicon sequencing	Patients with aMCI have a specific “GM‐intrinsic brain activity‐cognitive function” interaction pattern.
Verhaar et al.^60^	2021	AD (*n* = 33); MCI (*n* = 21); SCD (*n* = 116)	Structural MRI 16S rRNA amplicon sequencing	*Lachnospiraceae NK4A136 group spp*. and *Anaerostipes spp*. correlated with lower GCA visual scores.
He et al.^37^	2023	CI (*n* = 30); SCD (*n* = 62); control (*n* = 35)	Structural MRI 16S rRNA amplicon sequencing	*Mediterraneibacter* was significantly correlated with brain GMV and regional cortical structures (e.g., internal olfactory area, parahippocampal gyrus)

Abbreviations: AD, Alzheimer's disease; aMCI, amnestic mild cognitive impairment; CI, cognitive impairment; GCA, global cortical atrophy; GM, gut microbiota; GMV, gray matter volume; MCI, mild cognitive impairment; MRI, magnetic resonance imaging; rRNA, ribosomal RNA; SCD, subjective cognitive decline; VSRAD, voxel‐based specific regional analysis system for Alzheimer's disease.

NfL, as intermediate filament proteins of neuronal cytoskeleton, is related to axonal structure and function. Increased levels of NfL in CSF and plasma have emerged as a neuronal injury biomarker in neurodegenerative disorders. Previous studies have found that NfL is associated with AD and appears to provide information on disease progression and help monitor treatment effects.^84^, ^85^ In addition, NfL is also altered in other neurological diseases, such as cerebrovascular disease, frontotemporal dementia, Parkinson's disease, and amyotrophic lateral sclerosis.^86^, ^87^, ^88^, ^89^ Currently, there are only a few studies involving the correlation between the gut microbiota and NfL. Vogt et al. found that the gut microbial‐derived metabolite trimethylamine N‐oxide (TMAO) in the CSF is elevated in patients with MCI and AD dementia, which was positively associated with NfL.^90^ These findings suggest the involvement of the gut microbiota in the neurodegenerative process of AD. However, a recent study by Saji et al. reported that plasma NfL levels were not significantly correlated with gut microbial metabolites.^91^ Therefore, the effect of gut microbiota on the changes in NfL needs further evaluations.

#### 4.2.2 Gut microbiota and “I”

Glial fibrillary acidic protein (GFAP) is added to the revised criteria as an inflammation biomarker. GFAP is a hallmark of astrocytic activation. Astrocytes play an important role in the regulation of neuronal physiology, and their disintegration leads to the release of GFAP from brain tissues into the blood.^92^ Several recent studies have confirmed that plasma GFAP is higher in AD and may serve as a potential blood biomarker for AD.^93^, ^94^, ^95^ Increased plasma GFAP even has diagnostic and longitudinal monitoring potential for preclinical AD.^96^ Although specific bacterial genera are associated with immune markers,^97^ the effect of intestinal flora on the astrocytic activation (e.g., GFAP) in AD has not been widely explored. One study reported that after gut microbial perturbations by antibiotics and a germ‐free environment, the APPPS1‐21 mouse model of amyloidosis reduced GFAP+ reactive astrocytosis and astrocyte recruitment to amyloid plaques in male mice. Moreover, fecal microbiota from untreated APPPS1‐21 mice transplanted into antibiotics‐treated APPPS1‐21 mice restored astrocytic changes, suggesting that the gut microbiota can regulate GFAP+ astrocyte reactivity.^14^ These findings also provide new insights into the role of the gut–brain axis in AD.

### 4.3 Gut microbiota and non‐AD co‐pathologies biomarkers

#### 4.3.1 Gut microbiota and “V”

Biomarkers of vascular brain injury are derived from neuroimaging measures, including anatomic infarction, white matter hyperintensities (WMH). Several studies have revealed the potential relationship between the gut microbiota and vascular brain injury.^98^, ^99^, ^100^ For instance, Ma et al. found that patients with lacunar cerebral infarction showed increased relative abundance of genus *Lactobacillus*, *Streptococcus*, *Veillonella*, *Acidaminococcus*, *Bacillus*, *Peptoclostridium*, *Intestinibacter*, *Alloscardovia*, and *Cloacibacillus* but declined genus *Agathobacter* and *Lachnospiraceae_UCG‐004*.^98^ In another study, no significant correlation between gut microbiome profiles and brain WMH was observed in cognitively normal individuals.^21^ Notably, most studies involving the association of gut microbiota with these vascular brain injury biomarkers focus on cerebrovascular diseases, such as acute cerebral infarction, while their correlations in AD are still lacking. In the future, further evidence is needed.

#### 4.3.2 Gut microbiota and “S”

Aggregated α‐synuclein (αSyn) is the core component of cytoplasmic inclusions called Lewy bodies.^101^ Several neurodegenerative disorders exhibit this pathological feature, such as Parkinson's disease, dementia with Lewy bodies (DLB), AD, and so on. Increasing evidence has revealed that the gut microbiota is closely related to the αSyn pathology and can regulate motor deficits.^102^, ^103^ However, the assumption as to whether the effect of gut microbiota on αSyn will further influence the progress of AD is still unknown.

## 5 Gut microbiota‐related AD therapeutic strategies

### 5.1 Fecal microbiota transplantation

Table 4 shows the current microbiome‐mediated therapeutic strategies for AD. Fecal microbiota transplantation (FMT) is used to transfer the fecal components from a healthy donor to the recipient's intestine, which can directly alter the gut microbiota compositions.^104^ FMT has been successfully applied in treating *Clostridium difficile* infections, and may also be a potential microbiome modulation approach for AD intervention.^105^, ^106^ Recent studies suggest that FMT has shown promising results in reducing AD pathology in mouse models.^54^, ^107^ Sun et al. found that after transferring the fecal microbiota from healthy donors into 6‐month APP/ PS1 mice for 4 weeks, levels of Aβ and hyperphosphorylated tau were reduced.^107^ Similarly, Kim et al. showed that treating ADLPAPT mice from 2 to 6 months old with fecal microbiota from healthy donors reduced Aβ burden, tau phosphorylation, Iba1+microglia, GFAP+ astrocytes, and monocytes, leading to improved performance in behavioral tests.^54^ However, one study found that the transplantation of fecal microbiota in abx‐treated APPPS1‐21 mice increased the Aβ deposition and microglial activation.^50^

**TABLE 4:** Summary of gut microbiota‐related interventions in human AD.

Study	Study design	Participants	Intervention approach	Treatment duration	Main findings
**Probiotics intervention**					
Akbari et al. (2023) (2016)^74^	RCT	AD patients (*n* = 60)	Probiotic milk containing *L. acidophilus*, *L. casei*, *B. bifidum*, and *L. fermentum*	12 weeks	Improved MMSE scores but no changed oxidative stress and inflammation, fasting plasma glucose, and other lipid profiles.
Leblhuber et al. (2018)^75^	Single‐arm study	AD patients (*n* = 20)	*L. casei W56, L. lactis W19, L. acidophilus W22, B. lactis W52, L. paracasei W20, L. plantarum W62, B. lactis W51, B. bifidum W23 and L. salivarius W24*	4 weeks	Declined fecal zonulin concentrations and increased *Faecalibacterium prausnitzii*.
Agahi et al. (2018)^76^	RCT	AD patients (*n* = 60)	*Two types of capsules including either Lactobacillus fermentum, Lactobacillus* *plantarum, and Bifidobacterium lactis or Lactobacillus acidophilus*, *Bifidobacterium bifidum, and Bifidobacterium longum*.	12 weeks	The cognitive and biochemical (inflammatory and oxidative) indications in the patients with severe AD are insensitive to the probiotic supplementation.
Kobayashi et al. (2019)^77^	RCT	Subjective memory complaints (*n* = 121)	*B. breve A1*	12 weeks	Neuropsychological test scores significantly increased in both groups but there was no significant intergroup difference.
Kobayashi et al. (2019)^78^	Single‐arm study	MCI (*n* = 27)	*B. breve A1*	24 weeks	Improved cognitive function, mood status, and gastrointestinal symptoms.
Ton et al. (2020)^79^	Single‐arm study	AD patients (*n* = 16)	*Pasteurized milk with 4% kefir grains containing the species Acetobacter* *aceti, Acetobacter sp., L. delbrueckii*, *L. fermentum, L. fructivorans, Enterococcus faecium, Leuconostoc spp., L. kefiranofaciens, Candida famata, and Candida krusei*	90 days	Improved cognitive function, decreased inflammation and oxidative stress.
Asaoka et al. (2022)^69^	RCT	MCI (*n* = 130)	*B. breve A1*	24 weeks	Improved cognitive function on some subscales scores and suppressed brain atrophy progression.
Fei et al. (2023)^80^	RCT	MCI (*n* = 42)	*L. plantarum BioF‐228, L. lactis BioF‐224, B. lactis CP‐9, L. rhamnosus Bv‐77, L. johnsonii MH‐68, L. paracasei MP137, L. salivarius AP‐32, L. acidophilus TYCA06, L. lactisLY‐66, B. lactis HNO19, L. rhamnosus HNO01, L. paracasei GL‐156, B. animalis BB‐115, L. casei CS‐773, L. reuteri TSR332, L. fermentum TSF331, B. infantis BLI‐02, and L. plantarum CN2018*	12 weeks	Enhanced cognitive function and sleep quality.

FMT intervention					
Hazan et al. (2020)^81^	Case report	An 82‐year‐old AD patient	A single 300 mL FMT infusion using stool from the patient's 85‐year‐old wife	/	Rapid improvement in AD symptoms.
Park et al. (2021)^82^	Case report	A 90‐year‐old woman with AD	Fecal microbiota from a 27‐year‐old healthy man	/	Improved cognitive function in this AD patient.
Chen et al. (2023)^83^	Single‐arm study	MCI (*n* = 5)	Fecal microbiota capsules	/	FMT could maintain and improve cognitive function.

Abbreviations: AD, Alzheimer's disease; FMT, fecal microbiota transplantation; MCI, mild cognitive impairment; MMSE, Mini‐Mental State Examination; RCT, randomized controlled trial.

So far, three studies have reported the application of FMT in patients with AD and MCI.^81^, ^82^, ^83^ In one case, an 82‐year‐old male AD patient who received the transplantation of fecal microbiota from his 85‐year‐old wife showed improved memory function and emotion.^81^ Another case study reported a 90‐year‐old woman with AD and severe *Clostridium difficile* infection. After receiving FMT treatment from a healthy 27‐year‐old male donor, this AD patient showed significant amelioration in cognitive function, gut microbial diversity, and the production of SCFAs.^82^ However, these studies are only based on an individual observation or small samples, and more evidence is needed to validate the therapeutic potential of FMT in human AD.

In conclusion, although the FMT approach has shown a promising perspective in ameliorating the pathogenesis and cognitive performance of AD mouse models, more studies are essential before FMT serves as a non‐pharmacological intervention for AD.

### 5.2 Probiotics intervention

Probiotics supplementation is a simple and safe way to regulate the gut microbiota. The most common components of probiotics are *Lactobacillus and Bifidobacterium*.^108^ Some studies have shown that probiotics supplementation exhibits a certain effect on AD mouse models.^109^, ^110^, ^111^ Kaur et al. used a probiotic preparation called VSL#3 to treat 6‐month‐old APP^NL‐G‐F^ mice for 8 weeks.^110^ They found that VSL#3 altered the gut microbial compositions and blood metabolites, with increased abundance of *Clostridia*, *Lachnospiracea*, and *Akkermansia* and the level of SCFAs. However, there was no significant effect of the VSL#3 supplementation on Aβ, GFAP, Iba1, and the cellular proliferation marker Ki‐67. This finding may be due to the severe pathological changes in the 6‐month‐old APP^NL‐G‐F^ model. Thus, although VSL#3 altered the compositions of the gut microbiota and levels of serum metabolites, it may be too late to reverse the already formed pathological features.^110^, ^112^ On the contrary, Abdelhamid et al. found that treatment with a strain of *Bifidobacterium breve* for 4 months reduced Aβ, Iba1, and pro‐inflammatory cytokines, while it increased ADAM10 and synaptic proteins in 3‐month‐old APP^NL‐G‐F^ mice.^113^ Recently, one study showed that the supplementation of probiotic preparation SLAB51 significantly improved cognitive deficits, and reduced Aβ deposition and neuronal damage in transgenic AD mice.^114^ SLAB51 also enhanced antioxidant and neuroprotective effects by activating the Sirtuin‐1 pathway, which was helpful to recover cognitive and behavioral impairment. SLAB51 significantly altered the compositions of the gut microbiota in AD mice, with increased abundance of beneficial bacteria and decreased harmful bacteria.^115^ In addition, the combination of *L. acidophilus, L. fermentum, B. lactis*, and *B. longum* also improved learning ability and decreased the oxidative stress in rats injected with Aβ_1‐42_ in the hippocampus.^116^

The effect of probiotics supplementation on human AD patients has also been investigated.^75^, ^77^, ^78^, ^79^, ^80^ In a randomized, double‐blind, placebo‐controlled trial, AD patients were divided into the probiotic group and the placebo group. The results showed that compared to the placebo group, patients in the probiotic group had significantly improved cognitive performance, especially in executive function and memory. They also exhibited decreased inflammatory factors, insulin resistance, and blood lipids, which are common metabolic abnormalities in AD.^74^ Similarly, one meta‐analysis showed that compared to controls, individuals who received probiotics had significantly improved cognitive function, and decreased levels of malondialdehyde and high‐sensitivity C‐reactive protein.^117^ Moreover, MCI patients receiving short‐chain *Bifidobacterium breve A1* treatment also showed improved performance in some neuropsychological tests.^118^ However, other studies have shown contradictory results. Previously, AD patients aged 65 to 90 years old were randomly assigned to the probiotic group and the placebo group. After 12 weeks, this study found that there were no significant differences in the levels of pro‐inflammatory cytokines (tumor necrosis factor α and interleukin [IL]‐6) and anti‐inflammatory cytokine (IL‐10), as well as the oxidative factors (malondialdehyde and 8‐hydroxydeoxyguanosine) and the antioxidant factors (total antioxidant capacity, glutathione) between the placebo group and the probiotic group.^76^ In addition, this study also found that compared to the placebo group, there was no significant improvement in cognitive performance in the probiotic group, suggesting that patients in the late stage of AD may be insensitive to the probiotic supplementation.^76^

In summary, current findings suggest that probiotics supplementation may provide a potential clinical value in the treatment of AD. However, it's worth noting that there is a great deal of variability in the formulation, dose, and treatment patterns of probiotics in these studies, possibly causing different intervention effects on AD. Additionally, individual heterogeneity in the severity of diseases may also affect the efficacy of probiotics. Thus, more large‐scale, consistent, and standardized studies are needed to determine the best probiotics treatment strategies for AD.

## 6 CURRENT ISSUES AND FUTURE DIRECTIONS

### 6.1 Standardized protocol for study design and data processing

Although most studies have revealed significant changes in gut taxonomic compositions and diversity both in human and mouse models of AD, several remarkable issues still need to be discussed. First, many studies are single center and lack validation of findings in another center/cohort, which will reduce the robustness of results. Remarkably, there is inconsistency in the altered microbial species reported in those published studies. For instance, the changing trend of phyla *Firmicutes* and *Bacteroidetes* in AD patients is inverse in some different studies. We concluded that these discrepancies may be due to numerous factors, such as small sample size, race, lifestyle difference, regional disparity, and so on. Second, the diagnostic criteria for AD are not consistent among these studies. The severity of AD may be heterogeneous. Patients in some studies appear to be have AD dementia, while other studies may include moderate or severe patients with AD dementia. In addition, only a few studies diagnose patients based on the AD‐related biomarkers and the heterogeneity of participants is relatively great among different studies. Thus, AD biomarkers should be included for precise diagnosis in further validating studies. Third, the fecal sample collection, data preprocessing, microbiome sequencing, and analysis methods (e.g., 16S amplicon and metagenomic sequencing) are also different in these research studies. A practical and reproducible microbiome analysis guide for AD would be helpful. Therefore, given that many factors impact the gut microbiota characteristics, the establishment of a unified and standardized protocol in the study design and data processing is necessary for future AD microbiome studies.

### 6.2 Potential modifiers of the relationship between the gut microbiome and AD

**FIGURE 3:**
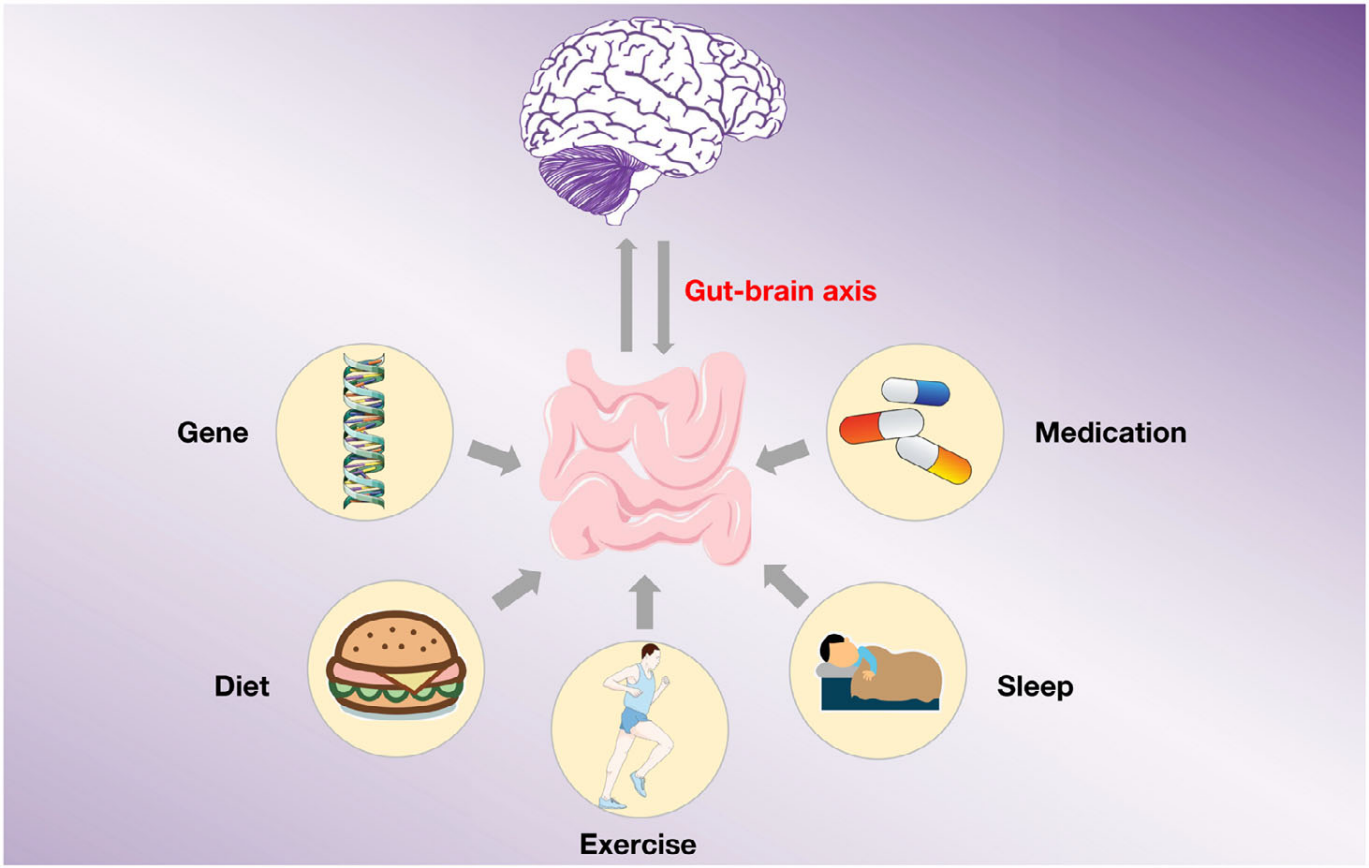
Potential factors linking to the gut microbiota and the “gut–brain axis.”

Many intrinsic and environmental factors, such as host genetics, lifestyles, and drugs, could influence the composition and diversity of the gut microbiome, which might mediate the correlation between the gut microbiome and AD pathogenesis. In this review, we discuss the association of these influence factors with the gut microbiome (Figure 3).

#### 6.2.1 Apolipoprotein E genotype

The apolipoprotein E (*APOE*) genotype is the primary genetic risk factor for AD. Currently, several studies have investigated the impact of *APOE* genotype on the gut microbiome structure and function.^119^, ^120^ Tran et al.^119^ detected that *APOE* genotype was associated with specific gut microbiome profiles in both humans and mice. Higher abundance of *Ruminococcaceae* and lower abundance of *Prevotellaceae* were observed in *APOE* ε2/ε3 than in other *APOE* genotypes. In addition, the *APOE* genotype can also influence the correlation between the gut microbiota and AD. For instance, pro‐inflammatory gut microbiota, such as genus *Collinsella*, may promote AD development through interaction with *APOE*.^121^ Furthermore, emerging evidence suggests that the *APOE* genotype mediates the effect of the gut microbiota on AD pathogenesis. A recent study also revealed that gut microbiota manipulations influenced tau pathology and neurodegeneration in an *APOE* genotype–dependent manner.^122^

#### 6.2.2 Lifestyles

Lifestyles might modify an individual's risk of developing AD.^123^, ^124^ Accumulating evidence suggests the potential impact of lifestyles, such as diet, exercise, and sleep, on the gut microbiota balances in health and disease.^125^, ^126^, ^127^, ^128^ Thus, we speculate that gut microbiota serve as mediators of lifestyle effects on AD development. Different dietary patterns may regulate the abundance of different gut microbiota, which further affect the pathogenesis of AD via different ways.^129^ For instance, a Western‐type dietary pattern increases the onset risk of AD, possibly by the mediation of the microbiota‐produced TMAO.^130^ Adherence to the Mediterranean diet may induce gut microbial changes, which is linked to the reduced risk of AD.^131^ In addition, calcium‐ and vegetable‐rich diets are also associated with the gut microbiota that produces SCFAs.^120^ SCFAs, as the most common form of gut microbiota–derived metabolites, are significantly altered in AD patients and animal models.^132^

There is compelling evidence that exercise is a protective intervention for AD. However, the mechanisms of the beneficial role of exercise on AD are not well understood. Several recent studies have focused on the modulatory effect of exercise on gut microbiota.^133^ Mitchell et al.^133^ found that exercise was associated with increased butyrate producing bacteria and fecal butyrate concentrations independent of diet in rodents and humans. The potential mechanisms by which exercise modulates gut microbiota may be closely linked to a beneficial anti‐inflammatory role, leading to the reduced gut inflammation.^134^

A large number of studies have shown that gut dysbiosis may play a critical role in the onset and pathogenesis of sleep disorders.^127^, ^128^ Accumulated evidences in this field have reported that there is a bidirectional regulation between the gut microbiota and sleep behavior.^135^ On one hand, gut microbiota exert a key role for the maintenance of normal sleep. Many studies have found that abnormal sleep patterns and duration are generally accompanied by altered gut microbiota. On the other hand, sleep disorders always affect the gut microbial composition and diversity. The potential mechanisms may be due to the activation of the hypothalamic–pituitary–adrenal axis.^127^ In addition, gut dysbiosis and abnormal sleep are also associated with AD pathogenesis.^136^

#### 6.2.3 Medication

Individuals display various responses to the treatment efficacy and adverse effect of medications. Increasing evidence suggests that the gut microbiome is responsible for this variability.^137^ There is a complex bidirectional interaction between the gut microbiome and commonly used medications.^138^ Several studies have reported the potential influence of specific medications on gut microbial composition and functional profiles. For instance, long‐term broad‐spectrum combinatorial antibiotic treatment could induce perturbations in gut microbial composition and diversity.^47^, ^48^ In addition to antibiotics, use of non‐antibiotic drugs, such as metformin,^139^, ^140^, ^141^ are also associated with altered gut microbiota. At the same time, the homeostasis in the gut ecosystem may directly and indirectly impact an individual's response to a specific drug.^142^ Understanding how the gut microbiome interacts with medications will further optimize the treatment efficacy.

## 7 CONCLUSIONS

The gut microbiota is a very important regulator for the development of AD. There are specific alterations in the gut microbial compositions in different stages of AD, including preclinical AD, MCI, and AD dementia. In addition, the gut microbiota exhibits close interaction with the pathogenesis of AD involving amyloidosis, tauopathy, neurodegeneration, inflammation, and co‐pathology. Notably, as the gut microbiota changes dynamically, their relationship with the progress of AD needs to be studied in prospective and longitudinal studies. In addition, it is of great importance to understand how the potential factors (e.g., sex, diet, sleep, drug) influence the gut microbiota, which may improve the therapeutic effect of gut microbiota‐based treatments in future clinical practice. In summary, understanding the causal relationship between the gut microbiota and AD pathophysiological features may contribute to the development of future drug targets for AD. Manipulating gut microbiota may be a promising avenue for the development of novel interventions for AD.

## CONFLICT OF INTEREST STATEMENT

The authors declare no competing interests. Author disclosures are available in the supporting information.

## Supporting information

Supporting Information

## References

[1] Scheltens P , De Strooper B , Kivipelto M , et al. Alzheimer's disease. Lancet. 2021;397:1577‐15790. 33667416 10.1016/S0140-6736(20)32205-4 PMC8354300

[2] 2023 Alzheimer's disease facts and figures . 2023 Alzheimer's disease facts and figures Alzheimers Dement. 2023;19:1598‐1695. 36918389 10.1002/alz.13016

[3] Dubois B , Villain N , Frisoni GB , et al. Clinical diagnosis of Alzheimer's disease: recommendations of the International Working Group. Lancet Neurol. 2021;20:484‐496. 33933186 10.1016/S1474-4422(21)00066-1 PMC8339877

[4] Karran E , De Strooper B . The amyloid hypothesis in Alzheimer disease: new insights from new therapeutics. Nat Rev Drug Discov. 2022;21:306‐318. 35177833 10.1038/s41573-022-00391-w

[5] Rabinovici GD , La Joie R . Amyloid‐targeting monoclonal antibodies for Alzheimer disease. JAMA. 2023;330:507‐509. 37459124 10.1001/jama.2023.11703

[6] Swanson CJ , Zhang Y , Dhadda S , et al. A randomized, double‐blind, phase 2b proof‐of‐concept clinical trial in early Alzheimer's disease with lecanemab, an anti‐Aβ protofibril antibody. Alzheimers Res Ther. 2021;13:80. 33865446 10.1186/s13195-021-00813-8 PMC8053280

[7] Tampi RR , Forester BP , Agronin M . Aducanumab: evidence from clinical trial data and controversies. Drugs Context. 2021;10. 10.7573/dic.2021-7-3 PMC8491638 34650610

[8] Sevigny J , Chiao P , Bussière T , et al. The antibody aducanumab reduces Aβ plaques in Alzheimer's disease. Nature. 2016;537:50‐56. 27582220 10.1038/nature19323

[9] Mintun MA , Lo AC , Duggan Evans C , et al. Donanemab in Early Alzheimer's Disease. N Engl J Med. 2021;384:1691‐1704. 33720637 10.1056/NEJMoa2100708

[10] Sims JR , Zimmer JA , Evans CD , et al. Donanemab in early symptomatic Alzheimer disease: the TRAILBLAZER‐ALZ 2 randomized clinical trial. JAMA. 2023;330:512‐527. 37459141 10.1001/jama.2023.13239 PMC10352931

[11] Ju Y , Tam KY . Pathological mechanisms and therapeutic strategies for Alzheimer's disease. Neural Regen Res. 2022;17:543‐549. 34380884 10.4103/1673-5374.320970 PMC8504384

[12] Janeiro MH , Ramírez MJ , Solas M . Dysbiosis and Alzheimer's disease: cause or treatment opportunity? Cell Mol Neurobiol. 2022;42:377‐387. 33400081 10.1007/s10571-020-01024-9 PMC11441293

[13] Shabbir U , Arshad MS , Sameen A , Oh DH . Crosstalk between gut and brain in Alzheimer's disease: the role of gut microbiota modulation strategies. Nutrients. 2021;13. 10.3390/nu13020690 PMC7924846 33669988

[14] Chandra S , Sisodia SS , Vassar RJ . The gut microbiome in Alzheimer's disease: what we know and what remains to be explored. Mol Neurodegener. 2023;18:9. 36721148 10.1186/s13024-023-00595-7 PMC9889249

[15] Hugon P , Dufour JC , Colson P , Fournier PE , Sallah K , Raoult D . A comprehensive repertoire of prokaryotic species identified in human beings. Lancet Infect Dis. 2015;15:1211‐1219. 26311042 10.1016/S1473-3099(15)00293-5

[16] Tarawneh R , Penhos E . The gut microbiome and Alzheimer's disease: complex and bidirectional interactions. Neurosci Biobehav Rev. 2022;141:104814. 35934087 10.1016/j.neubiorev.2022.104814 PMC9637435

[17] Cryan JF , O'Riordan KJ , Sandhu K , Peterson V , Dinan TG . The gut microbiome in neurological disorders. Lancet Neurol. 2020;19:179‐194. 31753762 10.1016/S1474-4422(19)30356-4

[18] Sochocka M , Donskow‐Łysoniewska K , Diniz BS , Kurpas D , Brzozowska E , Leszek J . The gut microbiome alterations and inflammation‐driven pathogenesis of Alzheimer's disease‐a critical review. Mol Neurobiol. 2019;56:1841‐1851. 29936690 10.1007/s12035-018-1188-4 PMC6394610

[19] Cattaneo A , Cattane N , Galluzzi S , et al. Association of brain amyloidosis with pro‐inflammatory gut bacterial taxa and peripheral inflammation markers in cognitively impaired elderly. Neurobiol Aging. 2017;49:60‐68. 27776263 10.1016/j.neurobiolaging.2016.08.019

[20] Cryan JF , O'Riordan KJ , Cowan CSM , et al. The microbiota‐gut‐brain axis. Physiol Rev. 2019;99:1877‐2013. 31460832 10.1152/physrev.00018.2018

[21] Ferreiro AL , Choi J , Ryou J , et al. Gut microbiome composition may be an indicator of preclinical Alzheimer's disease. Sci Transl Med. 2023;15:eabo2984. 37315112 10.1126/scitranslmed.abo2984 PMC10680783

[22] Sheng C , Yang K , He B , Du W , Cai Y , Han Y . Combination of gut microbiota and plasma amyloid‐β as a potential index for identifying preclinical Alzheimer's disease: a cross‐sectional analysis from the SILCODE study. Alzheimers Res Ther. 2022;14:35. 35164860 10.1186/s13195-022-00977-x PMC8843023

[23] Liu P , Wu L , Peng G , et al. Altered microbiomes distinguish Alzheimer's disease from amnestic mild cognitive impairment and health in a Chinese cohort. Brain Behav Immun. 2019;80:633‐643. 31063846 10.1016/j.bbi.2019.05.008

[24] Li B , He Y , Ma J , et al. Mild cognitive impairment has similar alterations as Alzheimer's disease in gut microbiota. Alzheimers Dement. 2019;15:1357‐1366. 31434623 10.1016/j.jalz.2019.07.002

[25] Vogt NM , Kerby RL , Dill‐McFarland KA , et al. Gut microbiome alterations in Alzheimer's disease. Sci Rep. 2017;7:13537. 29051531 10.1038/s41598-017-13601-y PMC5648830

[26] Sheng C , Lin L , Lin H , Wang X , Han Y , Liu SL . Altered gut microbiota in adults with subjective cognitive decline: the SILCODE study. J Alzheimers Dis. 2021;82:513‐526. 34024839 10.3233/JAD-210259

[27] Manderino L , Carroll I , Azcarate‐Peril MA , et al. Preliminary evidence for an association between the composition of the gut microbiome and cognitive function in neurologically healthy older adults. J Int Neuropsychol Soc. 2017;23:700‐705. 28641593 10.1017/S1355617717000492 PMC6111127

[28] Zhuang ZQ , Shen LL , Li WW , et al. Gut microbiota is altered in patients with Alzheimer's disease. J Alzheimers Dis. 2018;63:1337‐1346. 29758946 10.3233/JAD-180176

[29] Saji N , Niida S , Murotani K , et al. Analysis of the relationship between the gut microbiome and dementia: a cross‐sectional study conducted in Japan. Sci Rep. 2019;9:1008. 30700769 10.1038/s41598-018-38218-7 PMC6353871

[30] Liu P , Jia XZ , Chen Y , et al. Gut microbiota interacts with intrinsic brain activity of patients with amnestic mild cognitive impairment. CNS Neurosci Ther. 2021;27:163‐173. 32929861 10.1111/cns.13451 PMC7816203

[31] Guo M , Peng J , Huang X , Xiao L , Huang F , Zuo Z . Gut microbiome features of chinese patients newly diagnosed with Alzheimer's disease or mild cognitive impairment. J Alzheimers Dis. 2021;80:299‐310. 33523001 10.3233/JAD-201040

[32] Pan Q , Li YQ , Guo K , et al. Elderly patients with mild cognitive impairment exhibit altered gut microbiota profiles. J Immunol Res. 2021;2021:5578958. 34869782 10.1155/2021/5578958 PMC8635943

[33] Zhou Y , Wang Y , Quan M , Zhao H , Jia J . Gut microbiota changes and their correlation with cognitive and neuropsychiatric symptoms in Alzheimer's disease. J Alzheimers Dis. 2021;81:583‐595. 33814442 10.3233/JAD-201497

[34] Ling Z , Zhu M , Liu X , et al. Fecal fungal dysbiosis in chinese patients with Alzheimer's disease. Front Cell Dev Biol. 2020;8:631460. 33585471 10.3389/fcell.2020.631460 PMC7876328

[35] Ling Z , Zhu M , Yan X , et al. Structural and functional dysbiosis of fecal microbiota in chinese patients with Alzheimer's disease. Front Cell Dev Biol. 2020;8:634069. 33614635 10.3389/fcell.2020.634069 PMC7889981

[36] Cirstea MS , Kliger D , MacLellan AD , et al. The oral and fecal microbiota in a Canadian cohort of Alzheimer's disease. J Alzheimers Dis. 2022;87:247‐258. 35275538 10.3233/JAD-215520

[37] He B , Sheng C , Yu X , Zhang L , Chen F , Han Y . Alterations of gut microbiota are associated with brain structural changes in the spectrum of Alzheimer's disease: the SILCODE study in Hainan cohort. Front Aging Neurosci. 2023;15:1216509. 37520126 10.3389/fnagi.2023.1216509 PMC10375500

[38] McKhann G , Drachman D , Folstein M , Katzman R , Price D , Stadlan EM . Clinical diagnosis of Alzheimer's disease: report of the NINCDS‐ADRDA work group under the auspices of Department of health and human services task force on Alzheimer's Disease. Neurology. 1984;34:939‐944. 6610841 10.1212/wnl.34.7.939

[39] Dubois B , Feldman HH , Jacova C , et al. Research criteria for the diagnosis of Alzheimer's disease: revising the NINCDS‐ADRDA criteria. Lancet Neurol. 2007;6:734‐746. 17616482 10.1016/S1474-4422(07)70178-3

[40] Dubois B , Feldman HH , Jacova C , et al. Advancing research diagnostic criteria for Alzheimer's disease: the IWG‐2 criteria. Lancet Neurol. 2014;13:614‐629. 24849862 10.1016/S1474-4422(14)70090-0

[41] McKhann GM , Knopman DS , Chertkow H , et al. The diagnosis of dementia due to Alzheimer's disease: recommendations from the National Institute on Aging‐Alzheimer's Association workgroups on diagnostic guidelines for Alzheimer's disease. Alzheimers Dement. 2011;7:263‐269. 21514250 10.1016/j.jalz.2011.03.005 PMC3312024

[42] Sperling RA , Aisen PS , Beckett LA , et al. Toward defining the preclinical stages of Alzheimer's disease: recommendations from the National Institute on Aging‐Alzheimer's Association workgroups on diagnostic guidelines for Alzheimer's disease. Alzheimers Dement. 2011;7:280‐292. 21514248 10.1016/j.jalz.2011.03.003 PMC3220946

[43] Albert MS , DeKosky ST , Dickson D , et al. The diagnosis of mild cognitive impairment due to Alzheimer's disease: recommendations from the National Institute on Aging‐Alzheimer's Association workgroups on diagnostic guidelines for Alzheimer's disease. Alzheimers Dement. 2011;7:270‐279. 21514249 10.1016/j.jalz.2011.03.008 PMC3312027

[44] Jack CR, Jr. , Bennett DA , Blennow K , et al. NIA‐AA research framework: toward a biological definition of Alzheimer's disease. Alzheimers Dement. 2018;14:535‐562. 29653606 10.1016/j.jalz.2018.02.018 PMC5958625

[45] Jessen F , Amariglio RE , Buckley RF , et al. The characterisation of subjective cognitive decline. The Lancet Neurology. 2020;19:271‐278. 31958406 10.1016/S1474-4422(19)30368-0 PMC7062546

[46] Chen G , Zhou X , Zhu Y , Shi W , Kong L . Gut microbiome characteristics in subjective cognitive decline, mild cognitive impairment and Alzheimer's disease: a systematic review and meta‐analysis. Eur J Neurol. 2023. 10.1111/ene.15961 37399128

[47] Minter MR , Zhang C , Leone V , et al. Antibiotic‐induced perturbations in gut microbial diversity influences neuro‐inflammation and amyloidosis in a murine model of Alzheimer's disease. Sci Rep. 2016;6:30028. 27443609 10.1038/srep30028 PMC4956742

[48] Minter MR , Hinterleitner R , Meisel M , et al. Antibiotic‐induced perturbations in microbial diversity during post‐natal development alters amyloid pathology in an aged APP(SWE)/PS1(ΔE9) murine model of Alzheimer's disease. Sci Rep. 2017;7:10411. 28874832 10.1038/s41598-017-11047-w PMC5585265

[49] Dodiya HB , Kuntz T , Shaik SM , et al. Sex‐specific effects of microbiome perturbations on cerebral Aβ amyloidosis and microglia phenotypes. J Exp Med. 2019;216:1542‐1560. 31097468 10.1084/jem.20182386 PMC6605759

[50] Dodiya HB , Lutz HL , Weigle IQ , et al. Gut microbiota‐driven brain Aβ amyloidosis in mice requires microglia. J Exp Med. 2022;219. 10.1084/jem.20200895 PMC8647415 34854884

[51] Chen C , Ahn EH , Kang SS , Liu X , Alam A , Ye K . Gut dysbiosis contributes to amyloid pathology, associated with C/EBPβ/AEP signaling activation in Alzheimer's disease mouse model. Sci Adv. 2020;6 :eaba0466. 32832679 10.1126/sciadv.aba0466 PMC7439296

[52] Dodiya HB , Frith M , Sidebottom A , et al. Synergistic depletion of gut microbial consortia, but not individual antibiotics, reduces amyloidosis in APPPS1‐21 Alzheimer's transgenic mice. Sci Rep. 2020;10:8183. 32424118 10.1038/s41598-020-64797-5 PMC7235236

[53] Harach T , Marungruang N , Duthilleul N , et al. Reduction of Abeta amyloid pathology in APPPS1 transgenic mice in the absence of gut microbiota. Sci Rep. 2017;7:41802. 28176819 10.1038/srep41802 PMC5297247

[54] Kim MS , Kim Y , Choi H , et al. Transfer of a healthy microbiota reduces amyloid and tau pathology in an Alzheimer's disease animal model. Gut. 2020;69:283‐294. 31471351 10.1136/gutjnl-2018-317431

[55] Uchida Y , Kan H , Sakurai K , Oishi K , Matsukawa N . Contributions of blood‐brain barrier imaging to neurovascular unit pathophysiology of Alzheimer's disease and related dementias. Front Aging Neurosci. 2023;15:1111448. 36861122 10.3389/fnagi.2023.1111448 PMC9969807

[56] Uchida Y , Kan H , Sakurai K , et al. APOE ɛ4 dose associates with increased brain iron and β‐amyloid via blood‐brain barrier dysfunction. J Neurol Neurosurg Psychiatry. 2022. 10.1136/jnnp-2021-328519 35483916

[57] Megur A , Baltriukienė D , Bukelskienė V , Burokas A . The microbiota‐gut‐brain axis and Alzheimer's disease: neuroinflammation is to blame? Nutrients. 2020;13. 10.3390/nu13010037 PMC7824474 33374235

[58] Chen C , Liao J , Xia Y , et al. Gut microbiota regulate Alzheimer's disease pathologies and cognitive disorders via PUFA‐associated neuroinflammation. Gut. 2022. 10.1136/gutjnl-2021-326269 PMC10720732 35017199

[59] Friedland RP . Mechanisms of molecular mimicry involving the microbiota in neurodegeneration. J Alzheimers Dis. 2015;45:349‐362. 25589730 10.3233/JAD-142841

[60] Verhaar BJH , Hendriksen HMA , de Leeuw FA , et al. Gut microbiota composition is related to AD pathology. Front Immunol. 2021;12:794519. 35173707 10.3389/fimmu.2021.794519 PMC8843078

[61] Tetz G , Pinho M , Pritzkow S , Mendez N , Soto C , Tetz V . Bacterial DNA promotes Tau aggregation. Sci Rep. 2020;10:2369. 32047247 10.1038/s41598-020-59364-x PMC7012890

[62] Dominy SS , Lynch C , Ermini F , et al. Porphyromonas gingivalis in Alzheimer's disease brains: evidence for disease causation and treatment with small‐molecule inhibitors. Sci Adv. 2019;5:eaau3333. 30746447 10.1126/sciadv.aau3333 PMC6357742

[63] Zhan X , Stamova B , Jin LW , DeCarli C , Phinney B , Sharp FR . Gram‐negative bacterial molecules associate with Alzheimer disease pathology. Neurology. 2016;87:2324‐2332. 27784770 10.1212/WNL.0000000000003391 PMC5135029

[64] Saji N , Murotani K , Hisada T , et al. The relationship between the gut microbiome and mild cognitive impairment in patients without dementia: a cross‐sectional study conducted in Japan. Sci Rep. 2019;9:19227. 31852995 10.1038/s41598-019-55851-y PMC6920432

[65] Zhu J , Wang C , Qian Y , et al. Multimodal neuroimaging fusion biomarkers mediate the association between gut microbiota and cognition. Prog Neuropsychopharmacol Biol Psychiatry. 2022;113:110468. 34736997 10.1016/j.pnpbp.2021.110468

[66] Zhang S , Cai H , Wang C , Zhu J , Yu Y . Sex‐dependent gut microbiota‐brain‐cognition associations: a multimodal MRI study. BMC Neurol. 2023;23:169. 37106317 10.1186/s12883-023-03217-3 PMC10134644

[67] Zhang S , Xu X , Li Q , et al. Brain network topology and structural‐functional connectivity coupling mediate the association between gut microbiota and cognition. Front Neurosci. 2022;16:814477. 35422686 10.3389/fnins.2022.814477 PMC9002058

[68] Liang X , Fu Y , Cao WT , et al. Gut microbiome, cognitive function and brain structure: a multi‐omics integration analysis. Transl Neurodegener. 2022;11:49. 36376937 10.1186/s40035-022-00323-z PMC9661756

[69] Asaoka D , Xiao J , Takeda T , et al. Effect of probiotic bifidobacterium breve in improving cognitive function and preventing brain atrophy in older patients with suspected mild cognitive impairment: results of a 24‐week randomized, double‐blind, placebo‐controlled trial. J Alzheimers Dis. 2022;88:75‐95. 35570493 10.3233/JAD-220148 PMC9277669

[70] Portincasa P , Bonfrate L , Vacca M , et al. Gut microbiota and short chain fatty acids: implications in glucose homeostasis. Int J Mol Sci. 2022;23. 35163038 10.3390/ijms23031105 PMC8835596

[71] Wachsmuth HR , Weninger SN , Duca FA . Role of the gut‐brain axis in energy and glucose metabolism. Exp Mol Med. 2022;54:377‐392. 35474341 10.1038/s12276-021-00677-w PMC9076644

[72] Howard EJ , Lam TKT , Duca FA . The gut microbiome: connecting diet, glucose homeostasis, and disease. Annu Rev Med. 2022;73:469‐481. 34678047 10.1146/annurev-med-042220-012821

[73] Hao L , Wang L , Ju M , et al. 27‐Hydroxycholesterol impairs learning and memory ability via decreasing brain glucose uptake mediated by the gut microbiota. Biomed Pharmacother. 2023;168:115649. 37806088 10.1016/j.biopha.2023.115649

[74] Akbari E , Asemi Z , Daneshvar Kakhaki R , et al. Effect of probiotic supplementation on cognitive function and metabolic status in Alzheimer's disease: a randomized, double‐blind and controlled trial. Front Aging Neurosci. 2016;8:256. 27891089 10.3389/fnagi.2016.00256 PMC5105117

[75] Leblhuber F , Steiner K , Schuetz B , Fuchs D , Gostner JM . Probiotic supplementation in patients with Alzheimer's dementia ‐ an explorative intervention study. Curr Alzheimer Res. 2018;15:1106‐1113. 30101706 10.2174/1389200219666180813144834 PMC6340155

[76] Agahi A , Hamidi GA , Daneshvar R , et al. Does severity of Alzheimer's disease contribute to its responsiveness to modifying gut microbiota? A double blind clinical trial. Front Neurol. 2018;9:662. 30158897 10.3389/fneur.2018.00662 PMC6104449

[77] Kobayashi Y , Kuhara T , Oki M , Xiao JZ . Effects of Bifidobacterium breve A1 on the cognitive function of older adults with memory complaints: a randomised, double‐blind, placebo‐controlled trial. Benef Microbes. 2019;10:511‐520. 31090457 10.3920/BM2018.0170

[78] Kobayashi Y , Kinoshita T , Matsumoto A , Yoshino K , Saito I , Xiao JZ . Bifidobacterium breve A1 supplementation improved cognitive decline in older adults with mild cognitive impairment: an open‐label, single‐arm study. J Prev Alzheimers Dis. 2019;6:70‐75.30569089 10.14283/jpad.2018.32

[79] Ton AMM , Campagnaro BP , Alves GA , et al. Oxidative stress and dementia in Alzheimer's patients: effects of synbiotic supplementation. Oxid Med Cell Longev. 2020;2020:2638703. 32411323 10.1155/2020/2638703 PMC7201593

[80] Fei Y , Wang R , Lu J , et al. Probiotic intervention benefits multiple neural behaviors in older adults with mild cognitive impairment. Geriatr Nurs. 2023;51:167‐175. 36990042 10.1016/j.gerinurse.2023.03.006

[81] Hazan S . Rapid improvement in Alzheimer's disease symptoms following fecal microbiota transplantation: a case report. J Int Med Res. 2020;48:300060520925930. 32600151 10.1177/0300060520925930 PMC7328362

[82] Park SH , Lee JH , Shin J , et al. Cognitive function improvement after fecal microbiota transplantation in Alzheimer's dementia patient: a case report. Curr Med Res Opin. 2021;37:1739‐1744. 34289768 10.1080/03007995.2021.1957807

[83] Chen X , Zhang W , Lin Z , et al. Preliminary evidence for developing safe and efficient fecal microbiota transplantation as potential treatment for aged related cognitive impairments. Front Cell Infect Microbiol. 2023;13:1103189. 37113132 10.3389/fcimb.2023.1103189 PMC10127103

[84] Teunissen CE , Verberk IMW , Thijssen EH , et al. Blood‐based biomarkers for Alzheimer's disease: towards clinical implementation. The Lancet Neurology. 2022;21:66‐77. 34838239 10.1016/S1474-4422(21)00361-6

[85] Liu X , Chen J , Meng C , Zhou L , Liu Y . Serum neurofilament light chain and cognition decline in US elderly: A cross‐sectional study. Ann Clin Transl Neurol. 2023. 10.1002/acn3.51929 PMC10791034 37902309

[86] Gries M , Christmann A , Schulte S , et al. Parkinson mice show functional and molecular changes in the gut long before motoric disease onset. Mol Neurodegener. 2021;16:34. 34078425 10.1186/s13024-021-00439-2 PMC8170976

[87] Verde F , Otto M , Silani V . Neurofilament light chain as biomarker for amyotrophic lateral sclerosis and frontotemporal dementia. Front Neurosci. 2021;15:679199. 34234641 10.3389/fnins.2021.679199 PMC8255624

[88] Illán‐Gala I , Lleo A , Karydas A , et al. Plasma tau and neurofilament light in frontotemporal lobar degeneration and Alzheimer disease. Neurology. 2021;96:e671‐e683. 33199433 10.1212/WNL.0000000000011226 PMC7884995

[89] Peters N . Neurofilament light chain as a biomarker in cerebral small‐vessel disease. Mol Diagn Ther. 2022;26:1‐6. 34825310 10.1007/s40291-021-00566-y

[90] Vogt NM , Romano KA , Darst BF , et al. The gut microbiota‐derived metabolite trimethylamine N‐oxide is elevated in Alzheimer's disease. Alzheimer's Research & Therapy. 2018;10:124. 10.1186/s13195-018-0451-2 PMC6303862 30579367

[91] Saji N , Murotani K , Sato N , et al. Relationship between plasma neurofilament light chain, gut microbiota, and dementia: a cross‐sectional study. J Alzheimers Dis. 2022;86:1323‐1335. 35180112 10.3233/JAD-215141

[92] Heimfarth L , Passos FRS , Monteiro BS , Araújo AAS , Quintans Júnior LJ , Quintans JSS . Serum glial fibrillary acidic protein is a body fluid biomarker: a valuable prognostic for neurological disease ‐ A systematic review. Int Immunopharmacol. 2022;107:108624. 35255304 10.1016/j.intimp.2022.108624

[93] Verberk IMW , Thijssen E , Koelewijn J , et al. Combination of plasma amyloid beta((1‐42/1‐40)) and glial fibrillary acidic protein strongly associates with cerebral amyloid pathology. Alzheimers Res Ther. 2020;12:118. 32988409 10.1186/s13195-020-00682-7 PMC7523295

[94] Ganne A , Balasubramaniam M , Griffin WST , Shmookler Reis RJ , Ayyadevara S . Glial fibrillary acidic protein: a biomarker and drug target for Alzheimer's disease. Pharmaceutics. 2022;14. 35890250 10.3390/pharmaceutics14071354 PMC9322874

[95] Guo Y , Shen XN , Wang HF , et al. The dynamics of plasma biomarkers across the Alzheimer's continuum. Alzheimers Res Ther. 2023;15:31. 36750875 10.1186/s13195-023-01174-0 PMC9906840

[96] Chatterjee P , Pedrini S , Ashton NJ , et al. Diagnostic and prognostic plasma biomarkers for preclinical Alzheimer's disease. Alzheimer Demen: J Alzheimer's Assoc. 2022;18:1141‐1154. 10.1002/alz.12447 34494715

[97] Marizzoni M , Mirabelli P , Mombelli E , et al. A peripheral signature of Alzheimer's disease featuring microbiota‐gut‐brain axis markers. Alzheimers Res Ther. 2023;15:101. 37254223 10.1186/s13195-023-01218-5 PMC10230724

[98] Ma J , Xie H , Yuan C , et al. The gut microbial signatures of patients with lacunar cerebral infarction. Nutr Neurosci. 2023:1‐17. 37538045 10.1080/1028415X.2023.2242121

[99] Qian W , Wu M , Qian T , Xie C , Gao Y , Qian S . The roles and mechanisms of gut microbiome and metabolome in patients with cerebral infarction. Front Cell Infect Microbiol. 2023;13:1112148. 36761896 10.3389/fcimb.2023.1112148 PMC9905239

[100] Li H , Zhang X , Pan D , et al. Dysbiosis characteristics of gut microbiota in cerebral infarction patients. Transl Neurosci. 2020;11:124‐133. 33312718 10.1515/tnsci-2020-0117 PMC7706127

[101] Arnold MR , Coughlin DG , Brumbach BH , et al. α‐synuclein seed amplification in CSF and brain from patients with different brain distributions of pathological α‐synuclein in the context of co‐pathology and non‐LBD diagnoses. Ann Neurol. 2022;92:650‐662. 35808984 10.1002/ana.26453 PMC9489647

[102] Sampson TR , Debelius JW , Thron T , et al. Gut microbiota regulate motor deficits and neuroinflammation in a model of Parkinson's disease. Cell. 2016;167:1469‐1480.e12. 27912057 10.1016/j.cell.2016.11.018 PMC5718049

[103] Tan AH , Lim SY , Lang AE . The microbiome‐gut‐brain axis in Parkinson disease ‐ from basic research to the clinic. Nat Rev Neurol. 2022;18:476‐495. 35750883 10.1038/s41582-022-00681-2

[104] Gupta S , Allen‐Vercoe E , Petrof EO . Fecal microbiota transplantation: in perspective. Therap Adv Gastroenterol. 2016;9:229‐239. 10.1177/1756283X15607414 PMC4749851 26929784

[105] Rohlke F , Stollman N . Fecal microbiota transplantation in relapsing Clostridium difficile infection. Therap Adv Gastroenterol. 2012;5:403‐420. 10.1177/1756283X12453637 PMC3491681 23152734

[106] Nandwana V , Debbarma S . Fecal microbiota transplantation: a microbiome modulation technique for Alzheimer's disease. Cureus. 2021;13:e16503. 34430117 10.7759/cureus.16503 PMC8374998

[107] Sun J , Xu J , Ling Y , et al. Fecal microbiota transplantation alleviated Alzheimer's disease‐like pathogenesis in APP/PS1 transgenic mice. Translational Psychiatry. 2019;9:189. 31383855 10.1038/s41398-019-0525-3 PMC6683152

[108] Sanders ME , Merenstein DJ , Reid G , Gibson GR , Rastall RA . Probiotics and prebiotics in intestinal health and disease: from biology to the clinic. Nat Rev Gastroenterol Hepatol. 2019;16:605‐616. 31296969 10.1038/s41575-019-0173-3

[109] Abraham D , Feher J , Scuderi GL , et al. Exercise and probiotics attenuate the development of Alzheimer's disease in transgenic mice: Role of microbiome. Exp Gerontol. 2019;115:122‐131. 30529024 10.1016/j.exger.2018.12.005

[110] Kaur H , Golovko S , Golovko MY , Singh S , Darland DC , Combs CK . Effects of probiotic supplementation on short chain fatty acids in the AppNL‐G‐F mouse model of Alzheimer's disease. J Alzheimers Dis. 2020;76:1083‐1102. 32623399 10.3233/JAD-200436 PMC8104916

[111] Yang X , Yu D , Xue L , Li H , Du J . Probiotics modulate the microbiota‐gut‐brain axis and improve memory deficits in aged SAMP8 mice. Acta Pharm Sin B. 2020;10:475‐487. 32140393 10.1016/j.apsb.2019.07.001 PMC7049608

[112] Saito T , Matsuba Y , Mihira N , et al. Single App knock‐in mouse models of Alzheimer's disease. Nat Neurosci. 2014;17:661‐663. 24728269 10.1038/nn.3697

[113] Abdelhamid M , Zhou C , Ohno K , et al. Probiotic bifidobacterium breve prevents memory impairment through the reduction of both amyloid‐β production and microglia activation in APP knock‐in mouse. J Alzheimers Dis. 2022;85:1555‐1571. 34958017 10.3233/JAD-215025 PMC8925106

[114] Bonfili L , Cecarini V , Berardi S , et al. Microbiota modulation counteracts Alzheimer's disease progression influencing neuronal proteolysis and gut hormones plasma levels. Sci Rep. 2017;7. 10.1038/s41598-017-02587-2 PMC5445077 28546539

[115] Bonfili L , Cecarini V , Cuccioloni M , et al. SLAB51 probiotic formulation activates SIRT1 pathway promoting antioxidant and neuroprotective effects in an AD mouse model. Mol Neurobiol. 2018;55:7987‐8000. 29492848 10.1007/s12035-018-0973-4 PMC6132798

[116] Athari Nik Azm S , Djazayeri A , Safa M , et al. Lactobacilli and bifidobacteria ameliorate memory and learning deficits and oxidative stress in β‐amyloid (1‐42) injected rats. Appl Physiol Nutr Metab. 2018;43:718‐726. 29462572 10.1139/apnm-2017-0648

[117] Den H , Dong X , Chen M , Zou Z . Efficacy of probiotics on cognition, and biomarkers of inflammation and oxidative stress in adults with Alzheimer's disease or mild cognitive impairment ‐ a meta‐analysis of randomized controlled trials. Aging. 2020;12:4010‐4039. 32062613 10.18632/aging.102810 PMC7066922

[118] Xiao J , Katsumata N , Bernier F , et al. Probiotic Bifidobacterium breve in improving cognitive functions of older adults with suspected mild cognitive impairment: a randomized, double‐blind, placebo‐controlled trial. J Alzheimers Dis. 2020;77:139‐147. 32623402 10.3233/JAD-200488 PMC7592675

[119] Tran TTT , Corsini S , Kellingray L , et al. APOE genotype influences the gut microbiome structure and function in humans and mice: relevance for Alzheimer's disease pathophysiology. Faseb j. 2019;33:8221‐8231. 30958695 10.1096/fj.201900071R PMC6593891

[120] Hammond TC , Green SJ , Jacobs Y , et al. Gut microbiome association with brain imaging markers, APOE genotype, calcium and vegetable intakes, and obesity in healthy aging adults. Front Aging Neurosci. 2023;15:1227203. 37736325 10.3389/fnagi.2023.1227203 PMC10510313

[121] Cammann D , Lu Y , Cummings MJ , et al. Genetic correlations between Alzheimer's disease and gut microbiome genera. Sci Rep. 2023;13:5258. 37002253 10.1038/s41598-023-31730-5 PMC10066300

[122] Seo DO , O'Donnell D , Jain N , et al. ApoE isoform‐ and microbiota‐dependent progression of neurodegeneration in a mouse model of tauopathy. Science. 2023;379:eadd1236. 36634180 10.1126/science.add1236 PMC9901565

[123] Livingston G , Sommerlad A , Orgeta V , et al. Dementia prevention, intervention, and care. Lancet. 2017;390:2673‐2734. 28735855 10.1016/S0140-6736(17)31363-6

[124] Livingston G , Huntley J , Sommerlad A , et al. Dementia prevention, intervention, and care: 2020 report of the Lancet Commission. Lancet. 2020;396:413‐446. 32738937 10.1016/S0140-6736(20)30367-6 PMC7392084

[125] Campaniello D , Corbo MR , Sinigaglia M , et al. How diet and physical activity modulate gut microbiota: evidence, and perspectives. Nutrients. 2022;14. 35745186 10.3390/nu14122456 PMC9227967

[126] Perler BK , Friedman ES , Wu GD . The role of the gut microbiota in the relationship between diet and human health. Annu Rev Physiol. 2023;85:449‐468. 36375468 10.1146/annurev-physiol-031522-092054

[127] Matenchuk BA , Mandhane PJ , Kozyrskyj AL . Sleep, circadian rhythm, and gut microbiota. Sleep Med Rev. 2020;53:101340. 32668369 10.1016/j.smrv.2020.101340

[128] Wang Z , Wang Z , Lu T , et al. The microbiota‐gut‐brain axis in sleep disorders. Sleep Med Rev. 2022;65:101691. 36099873 10.1016/j.smrv.2022.101691

[129] Zhang M , Zhao D , Zhou G , Li C . Dietary pattern, gut microbiota, and Alzheimer's disease. J Agric Food Chem. 2020;68:12800‐12809. 32090565 10.1021/acs.jafc.9b08309

[130] Arrona Cardoza P , Spillane MB , Morales Marroquin E . Alzheimer's disease and gut microbiota: does trimethylamine N‐oxide (TMAO) play a role? Nutr Rev. 2022;80:271‐281. 33942080 10.1093/nutrit/nuab022

[131] Solch RJ , Aigbogun JO , Voyiadjis AG , et al. Mediterranean diet adherence, gut microbiota, and Alzheimer's or Parkinson's disease risk: a systematic review. J Neurol Sci. 2022;434:120166. 35144237 10.1016/j.jns.2022.120166

[132] Sheng C , Chu X , He Y , et al. Alterations in peripheral metabolites as key actors in Alzheimer's disease. Curr Alzheimer Res. 2023. 10.2174/1567205020666230825091147 37622711

[133] Mitchell CM , Davy BM , Hulver MW , Neilson AP , Bennett BJ , Davy KP . Does exercise alter gut microbial composition? A systematic review. Med Sci Sports Exerc. 2019;51:160‐167. 30157109 10.1249/MSS.0000000000001760

[134] Gubert C , Kong G , Renoir T , Hannan AJ . Exercise, diet and stress as modulators of gut microbiota: Implications for neurodegenerative diseases. Neurobiol Dis. 2020;134:104621. 31628992 10.1016/j.nbd.2019.104621

[135] Han M , Yuan S , Zhang J . The interplay between sleep and gut microbiota. Brain Res Bull. 2022;180:131‐146. 35032622 10.1016/j.brainresbull.2021.12.016

[136] Li Y , Shao L , Mou Y , Zhang Y , Ping Y . Sleep, circadian rhythm and gut microbiota: alterations in Alzheimer's disease and their potential links in the pathogenesis. Gut Microbes. 2021;13:1957407. 34520319 10.1080/19490976.2021.1957407 PMC8463034

[137] Zimmermann M , Zimmermann‐Kogadeeva M , Wegmann R , Goodman AL . Mapping human microbiome drug metabolism by gut bacteria and their genes. Nature. 2019;570:462‐467. 31158845 10.1038/s41586-019-1291-3 PMC6597290

[138] Weersma RK , Zhernakova A , Fu J . Interaction between drugs and the gut microbiome. Gut. 2020;69:1510‐1519. 32409589 10.1136/gutjnl-2019-320204 PMC7398478

[139] Wu H , Esteve E , Tremaroli V , et al. Metformin alters the gut microbiome of individuals with treatment‐naive type 2 diabetes, contributing to the therapeutic effects of the drug. Nat Med. 2017;23:850‐858. 28530702 10.1038/nm.4345

[140] Forslund K , Hildebrand F , Nielsen T , et al. Disentangling type 2 diabetes and metformin treatment signatures in the human gut microbiota. Nature. 2015;528:262‐266. 26633628 10.1038/nature15766 PMC4681099

[141] Mueller NT , Differding MK , Zhang M , et al. Metformin affects gut microbiome composition and function and circulating short‐chain fatty acids: a randomized trial. Diabetes Care. 2021;44:1462‐1471. 34006565 10.2337/dc20-2257 PMC8323185

[142] Vich Vila A , Collij V , Sanna S , et al. Impact of commonly used drugs on the composition and metabolic function of the gut microbiota. Nat Commun. 2020;11:362. 31953381 10.1038/s41467-019-14177-z PMC6969170

